# Sex and racial disparities in clinical outcomes and health resource use among patients with primary biliary cholangitis in the United States

**DOI:** 10.1097/HC9.0000000000001030

**Published:** 2026-09-28

**Authors:** Julius M. Wilder, Hongbo Yang, Su Zhang, Dongni Ye, Sherry Shi, Anyu Zhu, Ashley Jowell, Larissa Lushniak, Nisreen Shamseddine

**Affiliations:** 1 Duke University School of Medicine, Durham, North Carolina, USA; 2 Analysis Group, Inc., Boston, Massachusetts, USA; 3 Ipsen, Cambridge, Massachusetts, USA; * Affiliation at the time of this analysis and development of the manuscript

**Keywords:** cohort studies, health disparities, liver diseases, rare diseases, real-world data, retrospective studies

## Abstract

**Background:**

Racial and sex disparities have been described in liver conditions, including primary biliary cholangitis (PBC). This study sought to understand how race/ethnicity and sex-based differences impact access to diagnosis, clinical outcomes, health resource use (HRU), and treatment among patients with PBC in the United States (US).

**Methods:**

Komodo Research Database claims data (2016–2023) for adults diagnosed with PBC were analyzed retrospectively. Patients were grouped by race/ethnicity (White, Black, Hispanic, Asian, and Other) and separately by sex (male or female). Clinical characteristics, treatment patterns, and HRU outcomes were assessed pre-index and post-index. The index date was defined as the first claim with a PBC diagnosis code. Statistical significance for comparisons was defined as *p*<0.05.

**Results:**

In total, 9554 and 11,591 patients were included in the race/ethnicity and sex analyses. Black and Hispanic patients (versus [vs.] White) and male patients (vs. female) had higher comorbidity burden and PBC-related symptoms pre-index. Post-index, Black patients (vs. White) had significantly higher risks of liver transplant and hospitalization due to hepatic decompensation (adjusted hazard ratio [HR] of 2.09 and 1.37, respectively). Hispanic patients (vs. White) had a significantly higher risk of developing cirrhosis (adjusted HR of 1.19). Males had a higher risk than females for all adverse outcomes. Most HRU outcomes were significantly higher for Black patients vs. White, while treatment rates and response rates to ursodeoxycholic acid were significantly lower for Black patients vs. White and male patients vs. female.

**Conclusions:**

These results highlight significant differences in disease management and treatment initiation across race/ethnicity and sex groups in the US. Improved provider awareness of such differences in PBC is required to support individualized management and ensure equitable access to PBC treatment.

## Graphical abstract

**Figure:**
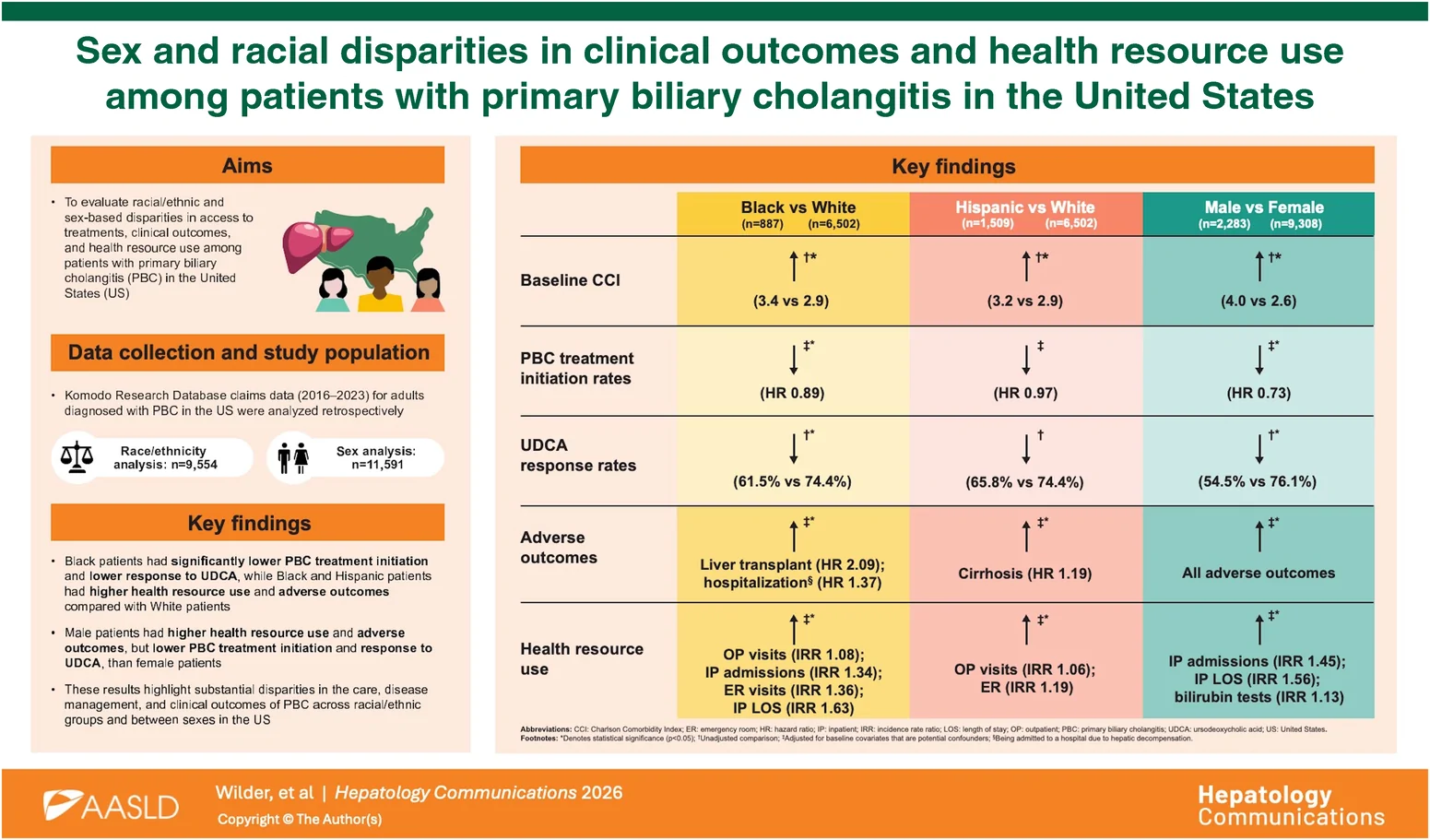

## INTRODUCTION

Primary biliary cholangitis (PBC) is a rare cholestatic liver disease that predominantly affects women aged 40–60 years.^1^^–^^3^ PBC is characterized by the autoimmune destruction of intrahepatic bile ducts, which can progress to cirrhosis, the need for liver transplantation (LT), and/or premature death if left untreated.^1^^,^^4^

Timely diagnosis and treatment of PBC is important since it can prevent or delay disease progression.^1^ To date, ursodeoxycholic acid (UDCA) is the recommended first-line (1L) treatment for patients with PBC per the 2017 European Association for the Study of the Liver (EASL) and the 2018 American Association for the Study of Liver Diseases (AASLD) treatment guidelines.^1^^,^^5^ Although some patients show liver biochemistry improvements following treatment, up to 40% do not achieve adequate response to UDCA,^6^^,^^7^ and 3%–5% of patients are intolerant.^8^ The United States (US) Food and Drug Administration (FDA) has approved second-line treatments: obeticholic acid (OCA) and 2 proliferator-activated receptor agonists, elafibranor and seladelpar (approved on June 10, 2024, and August 14, 2024, respectively).^9^^–^^12^ However, OCA was voluntarily withdrawn from the US market in September 2025 at the FDA’s request.^13^ Fibrates have been used as an off-label treatment for patients with an incomplete response to UDCA per the 2018 AASLD guideline recommendations; however, their use is contraindicated for patients with decompensated liver disease.^14^^,^^15^

In the US, the estimated prevalence of PBC is 40.9 per 100,000 persons.^16^ Women are more commonly affected than men (approximately 9:1) according to recent epidemiological studies, although estimates vary.^2^ PBC affects all races and ethnicities, while White patients are disproportionately more likely to be diagnosed,^17^ prevalence of diagnosis is increasing among other groups in the US, including Black and Asian individuals.^18^^,^^19^

The US has a diverse population, and racial and ethnic disparities in health outcomes are well documented.^20^ A 2025 systematic literature review by Faugno et al^21^ that included studies of patients across various health conditions found that patients from underserved racial and ethnic groups in the US (specifically Black, Native American/Alaskan Native, Hispanic, and Latino patients) encountered barriers to timely diagnosis stemming from socioeconomic and sociocultural factors (eg, trust in the health care system, health literacy barriers), provider-level factors (eg, cognitive biases, culturally sensitive communication), and health systems factors (eg, inequity in organizational health literacy, lack of cultural competence).

Race/ethnic and sex-based health disparities have previously been described in liver conditions, including PBC. Clinical trials in PBC are predominantly composed of White female patients; in 2 recent phase 3 trials investigating elafibranor and seladelpar, approximately 90% of participants were White and >90% were female.^10^^,^^22^ Male patients and patients from racial/ethnic groups that have been considered minority populations in the US, including Black or African American, Hispanic or Latino, and Asian patients, are not well represented in clinical trials despite also being affected by PBC.^23^ A previous study found that among patients with PBC on a LT waitlist, Hispanic patients had lower LT rates and a significantly higher risk of death than White patients.^24^ Previous studies also found that Hispanic patients had higher hospitalization rates and that Black patients had a higher in-hospital mortality rate than White patients.^18^^,^^23^ Limited but heterogenous data on PBC presentation and prognosis across sexes have also been reported in published literature. In some studies, male sex was a risk factor for mortality in PBC and associated with more advanced disease at presentation, poor response to 1L UDCA treatment, increased hepatocellular carcinoma (HCC) risk, and delayed diagnosis.^25^^–^^27^ In addition, a US real-world study of UDCA treatment found that men were significantly less likely than women to receive treatment, and African American patients were significantly less likely to receive treatment than White patients.^28^ However, other publications found no significant sex-based differences in outcomes or laboratory measures in PBC.^29^^,^^30^ These studies had limitations, including the use of historical data (collected ≥5 years to present day), non-US populations, and small sample sizes.

Therefore, there is a need to understand updated racial, ethnic, and sex-based disparities in PBC diagnosis and treatment. Greater provider education on inequities in treatment may lead to individualized management and equitable access to PBC treatment. Real-world evidence, including claims-based data, provides timely and representative insights into PBC care and clinical outcomes in the US and can be used to address this evidence gap and inform clinical decision-making.

The objective of this study was to understand how race/ethnicity and sex affect access to care, health care utilization, and clinical outcomes in PBC by conducting a retrospective analysis of the US claims data linked with laboratory data. This study is among the first to evaluate both race/ethnicity and sex disparities in PBC care and management.

## METHODS

### Study design

This observational, retrospective cohort study analyzed claims data from January 2016 to September 2023 using the Komodo Research Database (KRD+). The KRD+ contains administrative claims data on more than 330 million patients enrolled in US health care plans, including commercial, Medicare Advantage, and Medicaid managed care plans.^31^ Adult patients aged 18 years or above with a PBC diagnosis on or after January 1, 2017 were identified based on the International Classification of Diseases, Tenth Revision, Clinical Modification (ICD-10-CM) code K74.3. Patients were included if they had continuous enrollment in the database for ≥12 months before the first observed PBC diagnosis code and ≥1 month after that date, had not received LT or OCA before the first observed PBC diagnosis code, and had available race/ethnicity information (for the race/ethnicity analysis) or sex information (for the sex analysis). The index date was the date of the first claim associated with an ICD-10-CM code for PBC in the available data. The baseline period (pre-index) was defined as 12 months before the index date. The follow-up period was defined as time from index date to the earliest of death, end of continuous enrollment, or KRD+ data cutoff (henceforth referred to as post-index; Supplemental Figure S1, https://links.lww.com/HC9/C457). A cohort selection flowchart with sample selection criteria is presented in Supplemental Figure S2, https://links.lww.com/HC9/C457.

To assess race/ethnic disparities, eligible patients with race/ethnic group information were classified into 5 mutually exclusive cohorts as pre-defined in the KRD+: White, Black or African American (henceforth referred to as Black), Hispanic or Latino (henceforth referred to as Hispanic), Asian and Pacific Islanders (henceforth referred to as Asian), and other race/ethnic groups (henceforth referred to as Other). The race/ethnicity classification in the KRD+ used data from patients who had an outpatient (OP), inpatient (IP), or emergency room (ER) visit and who chose to report race/ethnicity. To assess sex disparities, eligible patients were classified as males and females based on biological sex, as reported in the KRD+.

### Study outcomes and statistical analysis

Patient characteristics, including patient demographics and clinical characteristics (comorbidities, PBC-related symptoms/complications, liver diseases resembling PBC, and health resource use [HRU] per-patient-per-year [PPPY]), were assessed during the pre-index period (Table 1). Comorbidities, PBC-related symptoms/complications, and liver diseases resembling PBC were determined using the ICD-10-CM diagnosis codes listed (Supplemental Table S1, https://links.lww.com/HC9/C457). These outcomes were descriptively summarized for each race/ethnicity group and for males and females, separately. Mean, standard deviation (SD), and range were used for continuous variables and counts and percentages (95% confidence interval [CI]) for categorical variables. Univariable comparisons were conducted to compare each non-White cohort versus (vs.) the White cohort and to compare males vs. females, respectively. *t* Test or Wilcoxon rank-sum test was conducted for continuous variables, and χ^2^ tests were conducted for categorical variables.

**TABLE 1:** Baseline characteristics for the race/ethnicity analysis cohort

	White	Black	Hispanic	Asian	Others
	N=6502	N=887	N=1509	N=342	N=314
Age as of index date (y), mean±SD	61.4±13.3	58.0±14.1*	57.4±13.1*	58.2±13.8*	56.5±13.2*
Female, n (%)	5099 (78.4)	702 (79.1)	1253 (83.0)*	249 (72.8)*	243 (77.4)
Insurance plan, n (%)					
Commercial	3347 (51.5)	408 (46.0)*	811 (53.7)*	207 (60.5)*	209 (66.6)*
Medicare	2684 (41.3)	351 (39.6)	456 (30.2)	97 (28.4)	80 (25.5)
Medicaid	465 (7.2)	125 (14.1)	240 (15.9)	38 (11.1)	25 (8.0)
Unknown	6 (0.1)	3 (0.3)	2 (0.1)	0 (0.0)	0 (0.0)
Geographic region, n (%)					
Northeast	2041 (31.4)	185 (20.9)*	294 (19.5)*	109 (31.9)*	108 (34.4)*
West	919 (14.1)	49 (5.5)	439 (29.1)	95 (27.8)	62 (19.7)
Midwest	1605 (24.7)	232 (26.2)	190 (12.6)	54 (15.8)	52 (16.6)
South	1937 (29.8)	421 (47.5)	585 (38.8)	84 (24.6)	92 (29.3)
Unknown	0 (0.0)	0 (0.0)	1 (0.1)	0 (0.0)	0 (0.0)
PBC-related symptoms and complications, n (%)					
Cirrhosis	1541 (23.7)	212 (23.9)	382 (25.3)	66 (19.3)	70 (22.3)
Clinically significant portal hypertension	1417 (21.8)	180 (20.3)	364 (24.1)	59 (17.3)*	64 (20.4)
Dyslipidemia	3444 (53.0)	482 (54.3)	769 (51.0)	172 (50.3)	153 (48.7)
Hepatic decompensation	1099 (16.9)	175 (19.7)*	257 (17.0)	58 (17.0)	53 (16.9)
End-stage liver disease	407 (6.3)	74 (8.3)*	115 (7.6)	21 (6.1)	22 (7.0)
HCC	51 (0.8)	16 (1.8)*	16 (1.1)	7 (2.0)*	3 (1.0)
Fatigue	1434 (22.1)	190 (21.4)	306 (20.3)	61 (17.8)	70 (22.3)
Liver fibrosis	280 (4.3)	49 (5.5)	89 (5.9)*	15 (4.4)	17 (5.4)
Pruritus	496 (7.6)	102 (11.5)*	121 (8.0)	29 (8.5)	22 (7.0)
Comorbidities, n (%)					
Gallstone disease (Cholelithiasis)	872 (13.4)	143 (16.1)*	213 (14.1)	43 (12.6)	42 (13.4)
Hypertension	3531 (54.3)	621 (70.0)*	812 (53.8)	153 (44.7)*	163 (51.9)
Osteoporosis	748 (11.5)	68 (7.7)*	149 (9.9)	47 (13.7)	25 (8.0)
Type 2 diabetes	1482 (22.8)	320 (36.1)*	490 (32.5)*	83 (24.3)	72 (22.9)
Urinary tract infection	938 (14.4)	141 (15.9)	261 (17.3)*	24 (7.0)*	44 (14.0)
Liver diseases and associated symptoms resembling PBC, n (%)					
Autoimmune hepatitis	521 (8.0)	105 (11.8)*	135 (8.9)	23 (6.7)	16 (5.1)
Primary sclerosing cholangitis	356 (5.5)	82 (9.2)*	43 (2.8)*	11 (3.2)	18 (5.7)
Inflammatory bowel disease	828 (12.7)	108 (12.2)	127 (8.4)*	22 (6.4)*	35 (11.1)
CCI, mean±SD	2.9±2.9	3.4±3.0*	3.2±2.9*	2.5±3.1*	2.9±2.8
No. ER visits (PPPY), mean±SD	1.1±4.7	2.0±6.0*	1.5±4.5*	0.9±3.7*	1.1±2.2
No. IP admissions (PPPY), mean±SD	0.4±1.0	0.6±2.0*	0.4±1.0	0.3±0.9*	0.4±0.9
Lab test results during baseline					
ALP, n (%)	1008 (15.5)	134 (15.1)	190 (12.6)*	57 (16.7)	37 (11.8)
Latest ALP (U/L), mean±SD	213.8±167.4	239.8±201.6	235.6±188.7	202.2±145.7	231.2±171.1
Latest ALP (U/L), median (IQR)	156.5 (106.0, 260.5)	159.5 (102.0, 314.0)	178.5 (106.0, 285.0)	147.0 (107.0, 239.0)	185.0 (111.0, 250.0)

*Notes:* (1) CCI was calculated based on Quan et al.^32^ (2) In comparing White vs.each of the other race groups, the *t* test is used for age at index, while the Wilcoxon rank-sum test is used for other continuous variables, given that their distributions are skewed; the χ^2^ test is used for categorical variables. (3) *Indicates a statistically significant difference (*p*<0.05). (4) Latest lab test values and duration from the latest lab result to the index date are summarized for those who have the corresponding test result available during the baseline period. The proportion of patients with ALP above normal is calculated among those who have ALP test results during baseline; the ULN of ALP is 120 IU/L.

Abbreviations: ALP, alkaline phosphatase; CCI, Charlson Comorbidity Index; ER, emergency room; HCC, hepatocellular carcinoma; IP, inpatient; IQR, interquartile range; LD, liver disease; PBC, primary biliary cholangitis; PPPY, per-patient-per-year; SD, standard deviation; ULN, upper limit of normal.

Multiple outcomes were assessed during the post-index period, including time to adverse clinical outcomes, time to first PBC treatment initiation (UDCA, OCA, and fibrates [fenofibrate or gemfibrozil]), all-cause HRU, time to specialist visits (visit to a hepatologist or gastroenterologist), and treatment response rate. PBC-related treatments were determined using the National Drug Code (Supplemental Table S2, https://links.lww.com/HC9/C457). Post-index adverse clinical outcomes of PBC included cirrhosis, clinically significant portal hypertension, hepatic decompensation, hospitalization due to hepatic decompensation, end-stage liver disease, HCC, LT, and death. These adverse clinical outcomes were identified using ICD-10-CM diagnosis codes provided in Supplemental Table S1, https://links.lww.com/HC9/C457. Time to each adverse clinical outcome and time to the earliest adverse clinical outcome (ie, earliest of any of the outcomes listed above) were evaluated using Kaplan-Meier (KM) analysis in patients who were free of the respective outcome during the pre-index period, with results stratified by cohort based on race/ethnicity (for the race/ethnicity analysis) and based on sex (for the sex analysis). Unadjusted log-rank tests were used to compare time-to-event outcomes across study groups. For adjusted time-to-event analyses, multivariable Cox proportional hazard (PH) models were used to adjust for baseline covariates that are potential confounders. Confounders were identified based on assessment of clinical relevance, statistical significance in baseline univariable comparisons, and the associated sample size (see Tables 2 and 3 for the list of confounders). For adverse clinical outcomes, Cox PH models were fitted to estimate HRs comparing each non-White cohort vs.the White cohort and comparing males vs.females, with and without adjusting for key baseline characteristics.

**TABLE 2:** Summary of adjusted hazard ratios for time to adverse clinical outcomes, time to first primary biliary cholangitis treatment, and time to first specialist visit during the post-index period by race/ethnicity based on Cox proportional hazard models

	Black	Hispanic	Asian	Others
	HR (95% CI)	HR (95% CI)	HR (95% CI)	HR (95% CI)
Adverse clinical outcomes				
Hepatic decompensation	1.20 (0.98, 1.47)	0.99 (0.82, 1.19)	0.97 (0.67, 1.40)	0.80 (0.53, 1.21)
IP due to hepatic decompensation	1.37 (1.06, 1.76)*	1.11 (0.88, 1.40)	1.06 (0.66, 1.70)	0.67 (0.38, 1.19)
Liver transplant	2.09 (1.40, 3.10)*	1.08 (0.72, 1.61)	2.15 (1.18, 3.91)*	0.94 (0.41, 2.15)
Cirrhosis	1.10 (0.93, 1.31)	1.19 (1.04, 1.37)*	0.98 (0.74, 1.28)	0.71 (0.50, 1.00)
Clinically significant portal hypertension	1.02 (0.84, 1.23)	1.08 (0.91, 1.27)	1.02 (0.76, 1.37)	0.77 (0.53, 1.13)
HCC	1.25 (0.71, 2.21)	1.44 (0.95, 2.19)	1.50 (0.69, 3.25)	1.22 (0.49, 3.00)
End-stage liver disease	1.19 (0.94, 1.51)	1.07 (0.87, 1.32)	1.25 (0.85, 1.83)	1.05 (0.69, 1.60)
All-cause death	1.09 (0.89, 1.35)	0.85 (0.70, 1.03)	0.39 (0.24, 0.64)*	1.48 (1.05, 2.08)*
Earliest of any clinical outcomes	0.98 (0.83, 1.16)	1.13 (0.99, 1.28)	0.89 (0.69, 1.15)	0.73 (0.54, 0.99)*
Time to first PBC treatment				
First PBC treatment	0.89 (0.82, 0.97)*	0.97 (0.91, 1.03)	0.97 (0.86, 1.10)	1.06 (0.93, 1.21)
Time to first specialist visit				
First specialist visit	0.98 (0.84, 1.15)	1.01 (0.89, 1.15)	1.11 (0.87, 1.41)	0.90 (0.70, 1.15)

*Notes:* (1) The multivariable models for time to adverse clinical outcomes and time to first PBC treatment adjusted for the following baseline variables: sex, age at index (65-y old or above or not), baseline CCI score (continuous), baseline liver diseases resembling PBC (autoimmune hepatitis [yes/no], PSC [yes/no], and IBD [yes/no]), baseline PBC symptoms/complications (cirrhosis [yes/no], clinically significant portal hypertension [yes/no], hepatic decompensation [yes/no], pruritus [yes/no], and fatigue [yes/no]; when a given condition was analyzed as the outcome, this condition was removed from the covariate adjustment), ever used PBC treatment (UDCA or fibrates) during baseline, baseline number of IP admissions (continuous), and baseline number of ER visits (continuous). When a given condition was analyzed as the outcome, this condition was removed from the covariate adjustment; when end-stage liver disease was the outcome, hepatic decompensation was also removed from the covariate adjustment because end-stage liver disease was a subcondition of hepatic decompensation. When the earliest of any clinical outcomes was the outcome, no PBC-related complications were adjusted for as covariates (ie, clinically significant portal hypertension, hepatic decompensation, cirrhosis, and pruritus). (2) The multivariable model for time to first special visit adjusted for the following baseline variables: sex (in racial/ethnic disparity only), race ethnicity (in sex disparity analysis only), age at diagnosis (65-y old or above or not), region at diagnosis, insurance plan type at diagnosis (commercial or not), baseline CCI score (continuous), baseline liver diseases resembling PBC (autoimmune hepatitis [yes/no], PSC [yes/no], and IBD [yes/no]), baseline PBC symptoms/complications (cirrhosis [yes/no], clinically significant portal hypertension [yes/no], hepatic decompensation [yes/no], pruritus [yes/no], and fatigue [yes/no]), ever used PBC treatment (UDCA or fibrates) during baseline, baseline number of IP admissions (continuous), and baseline number of ER visits (continuous). Patients with missing insurance plan type and region information were excluded from the multivariate models. (3) *Indicates a statistically significant difference (*p*<0.05).

Abbreviations: CCI, Charlson Comorbidity Index; CI, confidence interval; ER, emergency room; HCC, hepatocellular carcinoma; HR, hazard ratio; IBD, inflammatory bowel disease; IP, inpatient; PBC, primary biliary cholangitis; PSC, primary sclerosing cholangitis; UDCA, ursodeoxycholic acid.

**TABLE 3:** Summary of adjusted incidence rate ratios for all-cause health care resource use (per-patient-per-year) during the post-index period (race/ethnicity analysis)

	Black vs. White	Hispanic vs. White	Asian vs. White	Others vs. White
(A) No. medical visits				
** **Adjusted IRRs (95% CI)				
** **OP visits	1.08 (1.02, 1.13)*	1.06 (1.02, 1.10)*	0.96 (0.89, 1.04)	1.03 (0.95, 1.12)
** **ER visits	1.36 (1.24, 1.50)*	1.19 (1.09, 1.29)*	0.82 (0.69, 0.96)*	1.17 (1.00, 1.38)
** **IP admissions	1.34 (1.18, 1.54)*	0.93 (0.83, 1.04)	0.96 (0.77, 1.20)	0.90 (0.71, 1.14)
** **IP length of stay	1.63 (1.32, 2.02)*	1.02 (0.86, 1.22)	0.72 (0.52, 1.00)	0.58 (0.41, 0.82)*
(B) No. liver function lab tests				
** **Adjusted IRRs (95% CI)				
** **ALP tests	1.02 (0.90, 1.15)	0.90 (0.81, 0.99)*	1.11 (0.93, 1.33)	0.75 (0.62, 0.92)*
** **AST tests	1.00 (0.89, 1.13)	0.88 (0.80, 0.98)*	1.11 (0.93, 1.33)	0.72 (0.59, 0.89)*
** **ALT tests	1.02 (0.90, 1.15)	0.87 (0.79, 0.96)*	1.07 (0.90, 1.29)	0.70 (0.57, 0.86)*
** **Serum bilirubin tests	1.06 (0.95, 1.19)	0.94 (0.86, 1.03)	1.01 (0.85, 1.19)	0.80 (0.66, 0.96)*

*Notes:* (1) The multivariable models for number of medical visits/lab tests and time to first specialist visit adjusted for the following baseline variables: sex (in racial/ethnic disparity only), race ethnicity (in sex disparity analysis only), age at diagnosis (65-y old or above or not), region at diagnosis, insurance plan type at diagnosis (commercial or not), baseline CCI score (continuous), baseline liver diseases resembling PBC (autoimmune hepatitis [yes/no], PSC [yes/no], and IBD [yes/no]), baseline PBC symptoms/complications (cirrhosis [yes/no], clinically significant portal hypertension [yes/no], hepatic decompensation [yes/no], pruritus [yes/no], and fatigue [yes/no]), ever used PBC treatment (UDCA or fibrates) during baseline, baseline number of IP admissions (continuous), and baseline number of ER visits (continuous). Patients with missing insurance plan type and region information were excluded from the multivariate models. (2) *Indicates a statistically significant difference (*p*<0.05).

Abbreviations: ALP, alkaline phosphatase; ALT, alanine aminotransferase; AST, aspartate aminotransferase; CCI, Charlson Comorbidity Index; CI, confidence interval; ER, emergency room; HRU, health care resource use; IBD, inflammatory bowel disease; IP, inpatient; IRR, incidence rate ratio; OP, outpatient; PBC, primary biliary cholangitis; PPPY, per-patient-per-year; PSC, primary sclerosing cholangitis; UDCA, ursodeoxycholic acid.

Time to first PBC treatment initiation post-index was evaluated using the same approach as described above for time to adverse clinical outcomes: KM analyses with log-rank tests were applied for unadjusted comparisons, and multivariable Cox PH models adjusting for baseline covariates were used for the adjusted comparisons.

All-cause HRU post-index included the number of medical visits (including number of IP admissions, OP admissions, and ER visits), IP lengths of stay, number of liver functional lab tests, and time to specialist visits. PBC-related lab tests were determined from the KRD+ data using Current Procedural Terminology/Healthcare Common Procedure Coding System and ICD-10 Procedure Coding System provided in Supplemental Table S3, https://links.lww.com/HC9/C457. HRU outcomes were examined on a PPPY basis, and generalized linear models with and without controlling key baseline characteristics were fitted to estimate incidence rate ratios (IRRs). Time to specialist visits was evaluated using the same approach as described above for time to adverse clinical outcomes. Patients who visited specialists during the pre-index period were included in this analysis, with the event date being the index date.

Treatment response rate to 1L UDCA treatment was estimated in each cohort and was compared between each non-White cohort vs.the White cohort and between male and female patients. Inadequate response was defined as second-line treatment initiation (OCA or fibrates) within 12 months of UDCA initiation or failure to meet lab-based response criteria: the modified Toronto criteria (alkaline phosphatase [ALP] ≤1.67x upper limit of normal [ULN]) or the modified Primary Biliary Cholangitis to Improve Survival Estimation (POISE) criteria (ALP <1.67x ULN and bilirubin <ULN), where ULN was 120 IU/L for ALP and 1.2 mg/dL for bilirubin.^33^^,^^34^ For all comparisons in this study, statistical significance was defined as *p*<0.05.

The Komodo database was deidentified and complies with the patient requirements of the Health Insurance Portability and Accountability Act. Institutional Review Board review was not required for this study.

## RESULTS

### Racial/ethnic disparities

#### Sample identification and cohort creation

Overall, 9554 patients with PBC were identified for the race/ethnicity analysis, including 6502 in the White cohort, 887 in the Black cohort, 1509 in the Hispanic cohort, 342 in the Asian cohort, and 314 in the Other cohort (Supplemental Figure S2, https://links.lww.com/HC9/C457).

#### Pre-index

Black and Hispanic patients were significantly younger than White patients pre-index but had significantly higher comorbidity burden, as reflected by mean (SD) Charlson Comorbidity Index (CCI) values of 3.4 (3.0) and 3.2 (2.9) vs.2.9 (2.9), respectively. The Asian and Other cohorts were also significantly younger than the White cohort at index. Asian patients had a significantly lower comorbidity burden compared with White patients, as reflected by a mean (SD) CCI of 2.5 (3.1) vs.2.9 (2.9) and a mean (SD) ER visits PPPY of 0.9 (3.7) vs.1.1 (4.7), while the Other cohort had comparable baseline clinical characteristics to the White cohort (Table 1).

Additionally, Black and Hispanic patients had more PBC-related symptoms and complications pre-index. Specifically, Black patients had significantly higher rates of hepatic decompensation (19.7% vs. 16.9%), pruritus (11.5% vs. 7.6%), end-stage liver disease (8.3% vs. 6.3%), and HCC (1.8% vs. 0.8%) than White patients; Hispanic patients had significantly higher rates of liver fibrosis than White patients (5.9% vs. 4.3%). Compared with White patients, Black patients also had significantly higher mean (SD) IP admissions (PPPY) of 0.4 (1.0) vs.0.6 (2.0). Both Black and Hispanic patients incurred significantly higher mean (SD) ER visits PPPY of 2.0 (6.0) and 1.5 (4.5), respectively, compared to White patients (1.1 [4.7]). Differences in baseline ALP levels across racial/ethnic groups were not statistically significant (Table 1).

#### Post-index

##### Adverse clinical outcomes

Compared with White patients, Black patients had significantly higher risks of being admitted to a hospital due to hepatic decompensation and receiving LT (adjusted HR [95% CI] of 1.37 [1.06–1.76] and 2.09 [1.40–3.10], respectively), and Hispanic patients had significantly higher risks of developing cirrhosis (adjusted HR [95% CI] of 1.19 [1.04–1.37]) (Table 2).

Asian patients had similar risks of developing adverse clinical outcomes compared to White patients, with a few exceptions: the Asian cohort had a significantly lower risk of all-cause death (adjusted HR [95% CI] of 0.39 [0.24–0.64]) but a significantly higher risk of LT (adjusted HR [95% CI] of 2.15 [1.18–3.91]). Risks of developing adverse clinical outcomes were also similar between the Other cohort and White patients, although the Other cohort had a significantly higher risk of all-cause death (adjusted HR [95% CI] of 1.48 [1.05–2.08]) and a lower risk of any clinical outcome (adjusted HR [95% CI] of 0.73 [0.54–0.99]) (Table 2).

##### Health resource use

Compared with White patients, Black patients had significantly higher rates of OP visits, ER visits, IP admissions, and IP length of stay, while Hispanic patients had significantly higher rates of OP visits and ER visits, adjusting for key baseline characteristics. Asian patients had significantly lower rates of ER visits than White patients, and patients from the Other cohort had significantly shorter IP length of stay. PPPY liver functional tests were significantly lower among the Hispanic and Other cohorts vs.the White cohort, adjusting for key baseline characteristics (Table 3; unadjusted IRRs in Supplemental Table S4, https://links.lww.com/HC9/C457). Time to specialist visits was similar between non-White cohorts and the White cohort (Table 2).

While Black patients had a higher risk of developing PBC-related adverse clinical outcomes than White patients, their PBC treatment initiation rate was significantly lower (adjusted HR [95% CI] of 0.89 [0.82–0.97]) (Table 2). For Hispanic patients, PBC treatment initiation rate over time was significantly lower compared with White patients in only the unadjusted analysis (unadjusted HR [95% CI] of 0.93 [0.87–1.00]) (Supplemental Table S5, https://links.lww.com/HC9/C457). Time to first PBC treatment was not significantly different between the Asian and Other cohorts and the White cohort (Table 2).

##### Treatment response

Treatment response rate to 1L UDCA was assessed among 614 (8.5%) patients who initiated 1L UDCA treatment and had adequate lab measures based on the modified Toronto criteria and among 540 (7.5%) patients based on the modified POISE criteria. Compared with White patients, Black and Hispanic patients had lower response rates; the difference between Black and White patients based on second-line treatment initiation and the modified Toronto criteria was statistically significant in the unadjusted comparison (61.5% vs. 74.4%, respectively). Asian patients had higher treatment response rates compared with White patients based on the Toronto (81.8% vs. 74.4%) and modified POISE (78.9% vs. 65.2%) criteria, but the differences were not statistically significant. Response rates in the Other cohort were not significantly different from those in the White cohort (Toronto: 71.4% vs. 74.4%; POISE: 71.4% vs. 65.2%) (Table 4).

**TABLE 4:** Treatment response to first-line ursodeoxycholic acid (race/ethnicity analysis)

	White	Black	Hispanic	Asian	Others
Patients initiated UDCA, n	**4947**	**642**	**1133**	**260**	**239**
Toronto criteria (ALP ≤1.67x ULN)					
Patients with adequate lab results according to the Toronto criteria, n	453	52	73	22	14
Response rate, n (%)	337 (74.4)	32 (61.5)*	48 (65.8)	18 (81.8)	10 (71.4)
POISE criteria (ALP <1.67x ULN and bilirubin <ULN)					
Patients with adequate lab results according to the POISE criteria, n	396	43	68	19	14
Response rate, n (%)	258 (65.2)	25 (58.1)	40 (58.8)	15 (78.9)	10 (71.4)

*Notes:* (1) ULN was 120 IU/L for ALP and 1.2 mg/dL for bilirubin. (2) *Indicates a statistically significant difference (*p*<0.05).

Abbreviations: ALP, alkaline phosphatase; POISE, Primary Biliary Cholangitis to Improve Surrival Estimation; UDCA, ursodeoxycholic acid; ULN, upper limit of normal.

### Sex disparities

#### Sample identification and cohort creation

Overall, 11,591 patients with PBC were identified for the sex disparity analyses (Supplemental Figure S2, https://links.lww.com/HC9/C457), including 9308 females and 2283 males (Table 5).

**TABLE 5:** Baseline characteristics for the sex disparity analysis cohort

	Female	Male
	N=9308	N=2283
Age as of index date (y), mean±SD	58.6±12.8	57.9±15.2
Race/ethnicity, n (%)		
White	5099 (54.8)	1298 (56.9)*
Black or African American	702 (7.5)	167 (7.3)
Hispanic or Latino	1253 (13.5)	240 (10.5)
Asian or Pacific Islanders	249 (2.7)	83 (3.6)
Other	243 (2.6)	66 (2.9)
Unknown	1762 (18.9)	429 (18.8)
Insurance plan, n (%)		
Commercial	5629 (60.5)	1304 (57.1)*
Medicare	2869 (30.8)	766 (33.6)
Medicaid	796 (8.6)	210 (9.2)
Unknown	14 (0.2)	3 (0.1)
Geographic region as of index date, n (%)		
Northeast	2534 (27.2)	616 (27.0)
West	1471 (15.8)	366 (16.0)
Midwest	2150 (23.1)	518 (22.7)
South	3150 (33.8)	783 (34.3)
Unknown	3 (0.0)	0 (0.0)
PBC-related symptoms and complications, n (%)		
Cirrhosis	1714 (18.4)	917 (40.2)*
Clinically significant portal hypertension	1593 (17.1)	898 (39.3)*
Dyslipidemia	4577 (49.2)	1205 (52.8)*
Hepatic decompensation	1208 (13.0)	752 (32.9)*
End-stage liver disease	441 (4.7)	310 (13.6)*
HCC	52 (0.6)	59 (2.6)*
Fatigue	2009 (21.6)	479 (21.0)
Liver fibrosis	437 (4.7)	126 (5.5)
Pruritus	753 (8.1)	173 (7.6)
Comorbidities, n (%)		
Gallstone disease (Cholelithiasis)	1104 (11.9)	442 (19.4)*
Hypertension	4619 (49.6)	1366 (59.8)*
Osteoporosis	1011 (10.9)	90 (3.9)*
Type 2 diabetes	2040 (21.9)	702 (30.7)*
Urinary tract infection	1376 (14.8)	193 (8.5)*
Liver diseases and associated symptoms resembling PBC, n (%)		
Autoimmune hepatitis	815 (8.8)	166 (7.3)*
Primary sclerosing cholangitis	288 (3.1)	332 (14.5)*
Inflammatory bowel disease	826 (8.9)	508 (22.3)*
CCI, mean±SD	2.6±2.6	4.0±3.4*
No. ER visits (PPPY), mean±SD	1.1±4.6	1.3±2.9*
No. IP admissions (PPPY), mean±SD	0.3±0.9	0.7±1.3*
Lab test results during baseline		
ALP, n (%)	1194 (12.8)	274 (12.0)
Latest ALP (U/L), mean±SD	215.3±168.9	239.6±192.4
Latest ALP (U/L), median (IQR)	156.0 (107.0, 259.0)	174.0 (102.0, 309.0)

*Notes:* (1) CCI was calculated based on Quan et al.^32^ (2) In comparing male vs.female, *t* test is used for age at index, while the Wilcoxon rank-sum test is used for other continuous variables, given that their distributions are skewed, and χ^2^ test is used for categorical variables. (3) *Indicates a statistically significant difference (*p*<0.05). (4) Latest lab test values and duration from the latest lab result to the index date are summarized for those who have the corresponding test result available during the baseline period. The proportion of patients with ALP above normal is calculated among those who have ALP test results during baseline; the ULN of ALP is 120 IU/L.

Abbreviations: ALP, alkaline phosphatase; CCI, Charlson Comorbidity Index; ER, emergency room; HCC, hepatocellular carcinoma; IP, inpatient; IQR, interquartile range; PBC, primary biliary cholangitis; PPPY, per-patient-per-year; SD, standard deviation; ULN, upper limit of normal.

#### Pre-index

There was no significant difference in age between males and females pre-index. However, males had significantly higher comorbidity burden than females as reflected by mean (SD) CCI of 4.0 (3.4) vs.2.6 (2.6), higher mean (SD) ER visits PPPY of 1.3 (2.9) vs.1.1 (4.6), and higher mean (SD) IP admissions PPPY of 0.7 (1.3) vs.0.3 (0.9) at baseline. Additionally, males had significantly higher rates of most PBC-related symptoms and complications compared to females, specifically cirrhosis (40.2% vs. 18.4%), clinically significant portal hypertension (39.3% vs. 17.1%), dyslipidemia (52.8% vs. 49.2%), hepatic decompensation (32.9% vs. 13.0%), end-stage liver disease (13.6% vs. 4.7%), and HCC (2.6% vs. 0.6%). Differences in baseline ALP levels between males and females were not statistically significant (Table 5).

#### Post-index

##### Adverse clinical outcomes

Compared with females, males had significantly higher risks of developing all adverse clinical outcomes examined in this study, adjusting for key baseline characteristics (Table 6).

**TABLE 6:** Summary of adjusted hazard ratios for time to adverse clinical outcomes, time to first primary biliary cholangitis treatment, and time to first specialist visit during the post-index period by sex based on Cox proportional hazard models

	Male vs. female
	N=2283
Adverse clinical outcomes	
Hepatic decompensation	1.49 (1.30, 1.72)*
IP due to hepatic decompensation	1.68 (1.41, 2.00)*
Liver transplant	2.16 (1.68, 2.78)*
Cirrhosis	1.23 (1.09, 1.38)*
Clinically significant portal hypertension	1.77 (1.56, 2.02)*
HCC	1.54 (1.12, 2.13)*
End-stage liver disease	1.42 (1.23, 1.64)*
All-cause death	1.51 (1.33, 1.72)*
Earliest of any clinical outcomes	1.50 (1.34, 1.68)*
Time to first PBC treatment	
First treatment	0.73 (0.68, 0.77)*
Time to first specialist visit	
First specialist visit	0.94 (0.77, 1.14)

*Notes* *:* (1) The multivariable models for time to adverse clinical outcomes and time to first PBC treatment adjusted for the following baseline variables: race/ethnicity, age at index (65-y old or above or not), baseline CCI score (continuous), baseline liver diseases resembling PBC (autoimmune hepatitis [yes/no], PSC [yes/no], and IBD [yes/no]), baseline PBC symptoms/complications (cirrhosis [yes/no], clinically significant portal hypertension [yes/no], hepatic decompensation [yes/no], pruritus [yes/no], and fatigue [yes/no]; when a given condition was analyzed as the outcome, this condition was removed from the covariate adjustment), ever used PBC treatment (UDCA or fibrates) during baseline, baseline number of IP admissions (continuous), and baseline number of ER visits (continuous). When a given condition was analyzed as the outcome, this condition was removed from the covariate adjustment; when end-stage liver disease was the outcome, hepatic decompensation was also removed from the covariate adjustment because end-stage liver disease was a subcondition of hepatic decompensation. When the earliest of any clinical outcomes was the outcome, no PBC-related complications were adjusted for as covariates (ie, clinically significant portal hypertension, hepatic decompensation, cirrhosis, and pruritus). (2) The multivariable model for time to first special visit adjusted for the following baseline variables: sex (in racial/ethnic disparity only), race ethnicity (in sex disparity analysis only), age at diagnosis (65-y old or above or not), region at diagnosis, insurance plan type at diagnosis (commercial or not), baseline CCI score (continuous), baseline liver diseases resembling PBC (autoimmune hepatitis [yes/no], PSC [yes/no], and IBD [yes/no]), baseline PBC symptoms/complications (cirrhosis [yes/no], clinically significant portal hypertension [yes/no], hepatic decompensation [yes/no], pruritus [yes/no], and fatigue [yes/no]), ever used PBC treatment (UDCA or fibrates) during baseline, baseline number of IP admissions (continuous), and baseline number of ER visits (continuous). Patients with missing insurance plan type and region information were excluded from the multivariable models. (3) *Indicates a statistically significant difference (*p*<0.05).

Abbreviations: CCI, Charlson Comorbidity Index; ER, emergency room; HCC, hepatocellular carcinoma; IBD, inflammatory bowel disease; IP, inpatient; PBC, primary biliary cholangitis; PSC, primary sclerosing cholangitis; UDCA, ursodeoxycholic acid.

##### Health resource use

Compared with females, males had significantly higher rates of IP admissions (adjusted IRR [95% CI] of 1.45 [1.32–1.59]), IP length of stay (adjusted IRR [95% CI] of 1.56 [1.34–1.81]), and serum bilirubin tests (adjusted IRR [95% CI] of 1.13 [1.05, 1.22). Conversely, males had significantly lower rates of OP visits compared with females (adjusted IRR [95% CI] of 0.96 [0.93–0.99] (Table 7; unadjusted IRRs in Supplemental Table S6, https://links.lww.com/HC9/C457). The difference in time to first specialist visit was not statistically significant between males and females after adjusting for key baseline characteristics (unadjusted HRs in Supplemental Table S7, https://links.lww.com/HC9/C457). While males had higher hazards of developing adverse clinical outcomes than females, male PBC treatment rates during the post-index period were significantly lower than those of females (adjusted HR [95% CI] of 0.73 [0.68, 0.77]) (Table 6).

**TABLE 7:** Summary of adjusted incidence rate ratios for all-cause health care resource use (per-patient-per-year) during the post-index period (sex analysis)

	Male vs. female
(A) No. medical visits	
** **Adjusted IRRs (95% CI)	
** **OP visits	0.96 (0.93, 0.99)*
** **ER visits	1.02 (0.95, 1.09)
** **IP admissions	1.45 (1.32, 1.59)*
** **IP length of stay	1.56 (1.34, 1.81)*
(B) No. liver function lab tests	
** **Adjusted IRRs (95% CI)	
** **ALP tests	1.08 (0.99, 1.17)
** **AST tests	1.07 (0.99, 1.17)
** **ALT tests	1.08 (0.99, 1.17)
** **Serum bilirubin tests	1.13 (1.05, 1.22)*

*Notes:* (1) The multivariable models for number of medical visits/lab tests and time to first specialist visit adjusted for the following baseline variables: sex (in racial/ethnic disparity only), race ethnicity (in sex disparity analysis only), age at diagnosis (65-y old or above or not), region at diagnosis, insurance plan type at diagnosis (commercial or not), baseline CCI score (continuous), baseline liver diseases resembling PBC (autoimmune hepatitis [yes/no], PSC [yes/no], and IBD [yes/no]), baseline PBC symptoms/complications (cirrhosis [yes/no], clinically significant portal hypertension [yes/no], hepatic decompensation [yes/no], pruritus [yes/no], and fatigue [yes/no]), ever used PBC treatment (UDCA or fibrates) during baseline, baseline number of IP admissions (continuous), and baseline number of ER visits (continuous). Patients with missing insurance plan type and region information were excluded from the multivariable models. (2) *Indicates a statistically significant difference (*p*<0.05).

Abbreviations: ALP, alkaline phosphatase; ALT, alanine aminotransferase; AST, aspartate aminotransferase; CCI, Charlson Comorbidity Index; CI, confidence interval; ER, emergency room; IBD, inflammatory bowel disease; IP, inpatient; IRR, incidence rate ratio; OP, outpatient; PBC, primary biliary cholangitis; PSC, primary sclerosing cholangitis; UDCA, ursodeoxycholic acid.

##### Treatment response

Treatment response rate to 1L UDCA was assessed among 640 (7.2%) patients who initiated 1L UDCA treatment and had adequate lab measures based on the modified Toronto criteria and among 562 (6.4%) patients who had adequate lab measures based on the modified POISE criteria. Males had significantly lower response rates than females: 54.5% of males and 76.1% of females fulfilled the Toronto criteria; 42.2% of males vs.68.6% of females fulfilled the POISE criteria (Table 8).

**TABLE 8:** Treatment response to first-line ursodeoxycholic acid (sex analysis)

	Female	Male
Patients initiated UDCA, n	7426	1417
Toronto criteria (ALP ≤1.67x ULN)		
Patients with adequate lab results according to Toronto criteria, n	539	101
Response rate, n (%)	410 (76.1)	55 (54.5)*
POISE criteria (ALP <1.67x ULN and bilirubin <ULN)		
Patients with adequate lab results according to POISE criteria, n	472	90
Response rate, n (%)	324 (68.6)	38 (42.2)*

*Notes:* (1) ULN was 120 IU/L for ALP and 1.2 mg/dL for bilirubin. (2) *Indicates a statistically significant difference (*p*<0.05).

Abbreviations: ALP, alkaline phosphatase; POISE, Primary Biliary Cholangitis to Improve Survival Estimate; UDCA, ursodeoxycholic acid; ULN, upper limit of normal.

## DISCUSSION

This retrospective observational study is among the first to leverage a large, nationally representative US claims database to systematically evaluate real-world disparities in PBC care by race/ethnicity and sex. The findings of this study provide important new evidence that extends prior literature, revealing significant differences across racial groups and sexes in diagnosis, clinical outcomes, treatment initiation, and treatment response.

### Pre-index burden

The clinical burden associated with PBC was unevenly distributed; Black patients experienced significantly more pre-index complications than White patients, including hepatic decompensation, end-stage liver disease, and HCC, while Hispanic patients had significantly higher rates of liver fibrosis. Similarly, male patients exhibited more pre-index symptoms and complications than females, despite similar ages at index. This study hypothesizes that increased pre-index complications among Black, Hispanic, and male patients may reflect systemic barriers to PBC care, including lower provider suspicion for disease presentation, causing these patients to be diagnosed at a more advanced disease stage, and structural inequities that non-White populations face. Delayed diagnosis potentially reflects a lack of awareness of PBC by providers, which could be exacerbated by the fact that PBC is a rare disease and can be challenging for health care providers to recognize and differentiate from other liver conditions.^35^

### Post-index burden

After the index, race/ethnic disparities in clinical outcomes persisted. Compared with White patients, Black patients had significantly higher risks of hospitalization due to hepatic decompensation or LT, whereas Hispanic patients had a higher risk of developing new-onset cirrhosis. The race/ethnic disparities in clinical outcomes identified in this study are consistent with prior literature; previous retrospective cohort studies using PBC hospitalizations from the National Inpatient Sample reported higher in-hospital mortality among African American patients with PBC compared to White patients.^18^^,^^23^ In the current study, Black and Hispanic patients experienced more PBC-related complications post-index, yet reported lower treatment rates compared with White patients. These increased complications may reflect more advanced disease among Black and Hispanic patients compared with White patients.^36^

Sex differences in disease progression were also observed. Among patients who were free of the respective symptoms and complications at index, males had significantly higher risks of developing nearly all adverse clinical outcomes post-index than females. This finding supports previous research demonstrating worse outcomes among males than females with PBC.^28^^,^^37^^,^^38^ Previous studies have reported that male sex, compared to female sex, is associated with higher risks of mortality, advanced fibrosis, poorer prognosis, LT, and HCC development.^25^^,^^27^^,^^39^^,^^40^ These findings may reflect faster disease progression in males, different PBC presentation compared to females, or diagnostic delays that lead to more advanced disease at the time of treatment initiation among males.^41^ The current study did not evaluate the biologic mechanisms of the observed differences between sex, and further research is required to better understand the reasons for sex differences in PBC.

This study highlights the substantial undertreatment of PBC, with nearly a quarter of patients not initiating UDCA treatment within 30 months of diagnosis, despite it being recommended in guidelines as the 1L therapy for PBC. A US longitudinal cohort study also found that only 79% of patients with PBC had received UDCA.^42^

Moreover, the differences in treatment between race/ethnicity and sex groups in the current study show that treatment is also unequally distributed. Black patients were significantly less likely to initiate treatment than White patients; similarly, males were less likely to initiate treatment than females. A real-world cohort study by Lu et al^28^ similarly reported lower UDCA treatment rates among African American patients in the Fibrotic Liver Disease (FOLD) Consortium cohort compared with White patients.^28^ Reasons for nontreatment in the FOLD cohort were evaluated by Shamaa and colleagues; provider-level factors included lack of referral to specialists, lack of specialist knowledge, and attitudes leading to inadequate PBC recognition. Patient-level factors included competing health concerns, medication costs, UDCA intolerance due to adverse events, and lack of appropriate follow-up.^43^ Additional factors contributing to the lower likelihood of accessing health care among Black and Hispanic patients vs.White patients include systemic barriers, trust in the provider, and cultural stigma.^44^^,^^45^ Consequently, Black and Hispanic patients with PBC may access medical care less frequently or later in their symptom course, potentially reducing the likelihood and timeliness of receiving treatment. A similar pattern is present among sex; males in the United States are less likely than females to seek help from health care professionals, restricting their opportunities to receive medical support.^46^ The small sample sizes of the Asian (n=342) and Other (n=314) cohorts in the current study may limit the representativeness of the results for these populations.

Among patients who received 1L UDCA with adequate laboratory data, disparities in treatment response were observed across race/ethnicity and sex. Response rates were significantly lower in Black patients and numerically lower in Hispanic patients compared with White patients, and lower in males compared with females. Poor biochemical response to treatment is more frequently observed in patients with PBC who have more severe biochemical and histological features at baseline, such as higher baseline serum ALP levels; this may partly explain the differences in treatment response observed in this study.^6^ Although baseline ALP was available for a limited number of patients (n=1426 in the race/ethnicity analysis; n=1468 in the sex analysis), Hispanic patients demonstrated numerically higher ALP levels than White patients. These findings align with previous literature describing elevated liver biochemistry values, including ALP, and lower 1L UDCA response rates among Hispanic patients with PBC compared with non-Hispanic patients.^47^

Finally, disparities in HRU across race/ethnicity and sex followed similar patterns to those in clinical outcomes and treatment. Black and Hispanic patients incurred higher HRU (OP, ER, and IP visits) post-index compared with White patients. This is likely driven by more advanced disease, lower treatment rates, and/or lower UDCA treatment responses among Black and Hispanic patients.^36^ Post-index HRU among Black and Hispanic patients vs.White patients may reflect inequalities in clinical outcomes and disease severity. In general, Asian and Other patients incurred less HRU pre-index and post-index. While these variations could be linked to differences in disease severity or treatment rates compared with non-Asian patients, further research is needed to better understand this result. Males had higher rates of IP visits and bilirubin testing than females, which could be driven by more advanced baseline disease among men, necessitating closer disease monitoring.^25^ The higher HRU rates in Black and Hispanic and male patients vs.White and female patients, respectively, indicate a need for earlier PBC diagnosis and appropriate care to prevent symptom progression and subsequently fewer IP, OP, and ER visits.

### Strengths and limitations

The current study builds on the limited evidence base describing race/ethnic and sex differences in PBC by capturing longitudinal outcomes in a real-world setting. This study utilized a large claims database, including real-world data on approximately 330 million patients, which allowed for a timely, representative sample of patients with PBC in the United States. The study population captures patient demographics that are more diverse than those typically observed in clinical trials, which often have narrow eligibility criteria. Furthermore, the use of real-world data reflects patterns of medical encounters and HRU outside controlled settings, supporting the relevance of these findings for the PBC population in the United States.^48^ The use of closed claims allowed the identification of all medical encounters covered by the insurance provider, which enabled the study to comprehensively capture medical activities during the study period, allowing for accurate estimates of HRU and timing of events. Additionally, the claims database was linked to laboratory test results for a subset of patients, meaning that important prognosis factors for PBC could be measured, allowing for the assessment of UDCA treatment response rate. Our findings regarding UDCA treatment rates are broadly consistent with previous US real-world studies are broadly consistent with US real-world studies in patients with PBC.^28^^,^^47^ Although comparisons between patients treated with UDCA and patients not treated with UDCA were outside the scope of this analysis, our results highlight important treatment outcome differences across racial/ethnic and sex groups. Future studies evaluating outcomes associated with UDCA treatment may assess its impact to better understand the observed disparities in this population.

This study is subject to general limitations inherent to analyses of administrative claims data, including the potential for misclassification due to coding inaccuracies. Relying solely on ICD codes might result in underestimation of symptoms, conditions, and comorbidities, such as pruritus and fatigue, meaning the burden of disease may be misrepresented across the study population. As with any study utilizing claims data, the reliability of the data relies on the quality of external sources, such as records from health care providers, insurance plans, and hospital systems. Administrative delays or errors in inputting patient data may result in discrepancies between the actual date of clinical diagnosis and the date of the first claim associated with an ICD diagnosis code. Differences in comorbidity profiles between groups may have influenced all-cause HRU, and HRU cannot be definitively attributed to PBC rather than other comorbidities. The beneficiaries in KRD+ include individuals with health insurance and a health encounter, so the results from this study may not be generalizable to patients with PBC who have different access to care, such as uninsured and undiagnosed individuals. Not all patients enrolled in health insurance may be included in KRD+, so the population may not be representative of all patients with insurance. Additionally, the study was limited to capturing information for which insurance claims had been filed, which could omit a range of treatments, incidents, and symptoms that patients experience but are not documented with an insurance claim. Therefore, these data reflect patient interactions with the health care system rather than comprehensively illustrating patients’ experiences. Laboratory test results were only available for a limited number of patients in this study; therefore, caution should be taken when interpreting treatment response and related laboratory findings, as they may not be generalizable to patients without available lab data. Race/ethnicity information was only available for about 60% of Komodo Health enrollees, which limits the sample size and statistical power for each race/ethnicity cohort. In particular, the relatively small sample sizes of the Asian and Other cohorts may limit the representativeness of these findings; results for these groups should be interpreted with caution. Additionally, because race/ethnicity information in the KRD+ is classified into a single, mutually exclusive category, individuals with multiracial or multiethnic identities cannot be identified. Lastly, the current study did not explore the mechanisms that could explain differences in diagnosis or clinical outcomes across non-White cohorts, such as health care-seeking behavior.

## CONCLUSIONS

This real-world retrospective cohort study identified significant disparities in the care of patients with PBC in the United States with regard to disease diagnosis, treatment utilization, HRU, and clinical outcomes across race/ethnicity and sex. Lack of or delay in treatment initiation could contribute to the clinical burden of PBC and reinforce observed health inequalities in liver conditions within the United States.^1^ Recent advances in the PBC therapeutic landscape offer some effective treatment options and the potential to improve clinical outcomes.^19^ The findings from this study highlight the need for providers to consider race/ethnicity and sex differences, which can impact PBC prognosis and treatment response. Improved health care provider awareness is crucial to support timely diagnosis and ensure appropriate health care decision-making and tailored management approaches, which can improve outcomes across all patients, particularly non-White populations and males who are at higher risk of poor clinical outcomes. Further research is warranted to better understand the driving factors that could influence the receipt of treatment and the ability of providers to deliver optimal care for all patients.

## Supplementary Material

## References

[1] European Association for the Study of the L. EASL Clinical Practice Guidelines: The diagnosis and management of patients with primary biliary cholangitis. J Hepatol. 2017;67:145–172. 10.1016/j.jhep.2017.03.022 28427765

[2] Galoosian A Hanlon C Zhang J Holt EW Yimam KK. Clinical updates in primary biliary cholangitis: Trends, epidemiology, diagnostics, and new therapeutic approaches. J Clin Transl Hepatol. 2020;8:49–60. 10.14218/JCTH.2019.00049 PMC7132015 32274345

[3] Lv T Chen S Li M Zhang D Kong Y Jia J. Regional variation and temporal trend of primary biliary cholangitis epidemiology: A systematic review and meta-analysis. J Gastroenterol Hepatol. 2021;36:1423–1434. 10.1111/jgh.15329 33141955

[4] Lindor KD Bowlus CL Boyer J Levy C Mayo M. Primary biliary cholangitis: 2018 Practice Guidance from the American Association for the Study of Liver Diseases. Hepatology. 2019;69:394–419. 10.1002/hep.30145 30070375

[5] Lindor KD Bowlus CL Boyer J Levy C Mayo M. Primary biliary cholangitis: 2021 practice guidance update from the American Association for the Study of Liver Diseases. Hepatology. 2022;75:1012–1013. 10.1002/hep.32117 34431119

[6] Corpechot C Abenavoli L Rabahi N Chrétien Y Andréani T Johanet C. Biochemical response to ursodeoxycholic acid and long-term prognosis in primary biliary cirrhosis. Hepatology. 2008;48:871–877. 10.1002/hep.22428 18752324

[7] Corpechot C Chazouillères O Poupon R. Early primary biliary cirrhosis: Biochemical response to treatment and prediction of long-term outcome. J Hepatol. 2011;55:1361–1367. 10.1016/j.jhep.2011.02.031 21703194

[8] Invernizzi P Floreani A Carbone M Marzioni M Craxi A Muratori L. Primary biliary cholangitis: Advances in management and treatment of the disease. Dig Liver Dis. 2017;49:841–846. 10.1016/j.dld.2017.05.001 28546061

[9] Gilead. Gilead’s Livdelzi (seladelpar) granted accelerated approval for primary biliary cholangitis by U.S. FDA. Accessed October 29, 2024. https://www.gilead.com/news/news-details/2024/gileads-livdelzi-seladelpar-granted-accelerated-approval-for-primary-biliary-cholangitis-by-us-fda

[10] Hirschfield GM Bowlus CL Mayo MJ Kremer AE Vierling JM Kowdley KV. A Phase 3 Trial of seladelpar in primary biliary cholangitis. N Engl J Med. 2024;390:783–794. 10.1056/NEJMoa2312100 38381664

[11] Ipsen. Ipsen’s Iqirvo® receives U.S. FDA accelerated approval as a first-in-class PPAR treatment for primary biliary cholangitis. Accessed October 29, 2024. https://www.ipsen.com/press-releases/ipsens-iqirvo-receives-u-s-fda-accelerated-approval-as-a-first-in-class-ppar-treatment-for-primary-biliary-cholangitis/

[12] United States Food and Drug Administration. OCALIVA® (obeticholic acid) Prescribing information. 2024. Accessed November 14, 2024. https://www.accessdata.fda.gov/drugsatfda_docs/label/2022/207999s008lbl.pdf

[13] Intercept announces voluntary withdrawal of OCALIVA® for primary biliary cholangitis (PBC) from the US Market; US Clinical Trials Involving Obeticholic Acid Placed on Clinical Hold. 2025. Accessed September 11, 2025.https://www.interceptpharma.com/about-us/news/?id=3148535.

[14] Cançado GGL Braga MH Ferraz MLG Villela-Nogueira CA Terrabuio DRB Cançado ELR. Clinical features and treatment outcomes of primary biliary cholangitis in a highly admixed population. Ann Hepatol. 2022;27:100546. 10.1016/j.aohep.2021.100546 34600142

[15] United States Food and Drug Administration. FENOGLIDE prescribing information. Accessed October 29, 2024. https://www.accessdata.fda.gov/drugsatfda_docs/label/2019/022118s010lbl.pdf

[16] Levy C Buchanan-Peart K-A MacEwan JP Levine A Nair R Wheeler D. A nationwide study of primary biliary cholangitis prevalence, geographic distribution, and health care providers. Hepatol Commun. 2025;9:e0677. 10.1097/HC9.0000000000000677 PMC11999412 40227093

[17] Gazda J Drazilova S Janicko M Jarcuska P. The epidemiology of primary biliary cholangitis in European countries: A systematic review and meta-analysis. Can J Gastroenterol Hepatol. 2021;2021:9151525. 10.1155/2021/9151525 PMC8238597 34239845

[18] Adejumo AC Akhtar DH Dennis BB Cholankeril G Alayo Q Ogundipe OA. Gender and racial differences in hospitalizations for primary biliary cholangitis in the USA. Dig Dis Sci. 2021;66:1461–1476. 10.1007/s10620-020-06402-3 32535779

[19] Trivella J John BV Levy C. Primary biliary cholangitis: Epidemiology, prognosis, and treatment. Hepatol Commun. 2023;7:e0179. 10.1097/HC9.0000000000000179 PMC10241503 37267215

[20] Macias-Konstantopoulos WL Collins KA Diaz R Duber HC Edwards CD Hsu AP. Race, healthcare, and health disparities: A critical review and recommendations for advancing health equity. West J Emerg Med. 2023;24:906–918. 10.5811/westjem.58408 PMC10527840 37788031

[21] Faugno E Galbraith AA Walsh K Maglione PJ Farmer JR Ong MS. Experiences with diagnostic delay among underserved racial and ethnic patients: A systematic review of the qualitative literature. BMJ Qual Saf. 2025;34:190–200. 10.1136/bmjqs-2024-017506 PMC11839380 39496473

[22] Kowdley KV Bowlus CL Levy C Akarca US Alvares-da-Silva MR Andreone P. Efficacy and safety of elafibranor in primary biliary cholangitis. N Engl J Med. 2024;390:795–805. 10.1056/NEJMoa2306185 37962077

[23] Galoosian A Hanlon C Tana M Cheung R Wong RJ. Race/ethnicity and insurance-specific disparities in in-hospital mortality among adults with primary biliary cholangitis: Analysis of 2007-2014 national inpatient sample. Dig Dis Sci. 2020;65:406–415. 10.1007/s10620-019-05809-x 31489564

[24] Cholankeril G Gonzalez HC Satapathy SK Gonzalez SA Hu M Khan MA. Increased waitlist mortality and lower rate for liver transplantation in hispanic patients with primary biliary cholangitis. Clin Gastroenterol Hepatol. 2018;16:965–973.e2. 10.1016/j.cgh.2017.12.017 PMC7331901 29427734

[25] Lleo A Jepsen P Morenghi E Carbone M Moroni L Battezzati PM. Evolving trends in female to male incidence and male mortality of primary biliary cholangitis. Sci Rep. 2016;6:25906. 10.1038/srep25906 PMC4872151 27192935

[26] Floreani A Gabbia D De Martin S. Are gender differences important for autoimmune liver diseases? Life (Basel). 2024;14:500. 10.3390/life14040500 PMC11050899 38672770

[27] Abdulkarim M Zenouzi R Sebode M Schulz L Quaas A Lohse AW. Sex differences in clinical presentation and prognosis in patients with primary biliary cholangitis. Scand J Gastroenterol. 2019;54:1391–1396. 10.1080/00365521.2019.1683226 31692389

[28] Lu M Li J Haller IV Romanelli RJ VanWormer JJ Rodriguez CV. Factors associated with prevalence and treatment of primary biliary cholangitis in united states health systems. Clin Gastroenterol Hepatol. 2018;16:1333–1341.e6. 10.1016/j.cgh.2017.10.018 29066370

[29] Rubel LR Rabin L Seeff LB Licht H Cuccherini BA. Does primary biliary cirrhosis in men differ from primary biliary cirrhosis in women? Hepatology. 1984;4:671–677. 10.1002/hep.1840040418 6745856

[30] Nalbandian G Van de Water J Gish R Manns M Coppel RL Rudich SM. Is there a serological difference between men and women with primary biliary cirrhosis? Am J Gastroenterol. 1999;94:2482–2486. 10.1111/j.1572-0241.1999.01380.x 10484012

[31] Komodo Health. Komodo healthcare map. Accessed September 23, 2025. https://www.komodohealth.com/solutions/healthcare-map/

[32] Quan H Li B Couris CM Fushimi K Graham P Hider P. Updating and validating the Charlson comorbidity index and score for risk adjustment in hospital discharge abstracts using data from 6 countries. Am J Epidemiol. 2011;173:676–682. 10.1093/aje/kwq433 21330339

[33] Duke Medicine. Duke University Reference Laboratory Values. Duke University School of Medicine. Accessed November 2025. https://pathology.oit.duke.edu/normalLabs.html

[34] Kalas MA Chavez L Leon M Taweesedt PT Surani S. Abnormal liver enzymes: A review for clinicians. World J Hepatol. 2021;13:1688–1698. 10.4254/wjh.v13.i11.1688 PMC8637680 34904038

[35] Peloquiin S Shiffman ML Kowdley KV Mena E Gossard AA Fan L. S1988 Knowledge, skills, and confidence gaps in diagnosis of primary biliary cholangitis among gastroenterologists and advance practice providers practicing in gastroenterology or hepatology. ACG. 2024;119(10S):S1424.

[36] Peters MG Di Bisceglie AM Kowdley KV Flye NL Luketic VA Munoz SJ. Differences between Caucasian, African American, and Hispanic patients with primary biliary cirrhosis in the United States. Hepatology. 2007;46:769–775. 10.1002/hep.21759 PMC4167731 17654740

[37] Hirschfield GM Chazouillères O Cortez-Pinto H Macedo G de Lédinghen V Adekunle F. A consensus integrated care pathway for patients with primary biliary cholangitis: A guideline-based approach to clinical care of patients. Expert Rev Gastroenterol Hepatol. 2021;15:929–939. 10.1080/17474124.2021.1945919 34233565

[38] Martini F Balducci D Mancinelli M Buzzanca V Fracchia E Tarantino G. Risk stratification in primary biliary cholangitis. J Clin Med. 2023;12:5713. 10.3390/jcm12175713 PMC10488776 37685780

[39] John BV Aitcheson G Schwartz KB Khakoo NS Dahman B Deng Y. Male sex is associated with higher rates of liver-related mortality in primary biliary cholangitis and cirrhosis. Hepatology. 2021;74:879–891. 10.1002/hep.31776 33636012

[40] Natarajan Y Tansel A Patel P Emologu K Shukla R Qureshi Z. Incidence of hepatocellular carcinoma in primary biliary cholangitis: A systematic review and meta-analysis. Dig Dis Sci. 2021;66:2439–2451. 10.1007/s10620-020-06498-7 32743773

[41] Xie M Nyabanga CT Sehmbhi M Khalili NA Chon C Kamath A. Clinicopathological and imaging features of primary biliary cholangitis. medRxiv. 2022. 2022.04.29.22274511

[42] Lu M Zhou Y Haller IV Romanelli RJ VanWormer JJ Rodriguez CV. Increasing prevalence of primary biliary cholangitis and reduced mortality with treatment. Clin Gastroenterol Hepatol. 2018;16:1342–1350.e1. 10.1016/j.cgh.2017.12.033 29277621

[43] Shamaa O Ahmed A Rupp L Gordon S. Factors associated with lack of PBC evaluation and treatment: A single tertiary care center experience. ACG. 2021;116:S41.

[44] Dwyer Orr L Sadik K Beusterien K Loomer S King-Concialdi K Krupsky K. Drivers and barriers to health-seeking behaviors and interactions: A qualitative study of Black patients with lung cancer and with peripheral artery disease. Curr Med Res Opin. 2025;41:135–143. 10.1080/03007995.2024.2444420 39819222

[45] Lin C Mella-Velazquez A Braund WE Tu P. Disparities in healthcare-seeking behavior and decision preference among Hispanics: A comparative study across races/ethnicities, SES, and provider types. J Multidiscip Healthc. 2024;17:3849–3862. 10.2147/JMDH.S476285 PMC11319094 39139698

[46] Galdas PM Cheater F Marshall P. Men and health help-seeking behaviour: Literature review. Journal of Advanced Nursing. 2005;49:616–623. 10.1111/j.1365-2648.2004.03331.x 15737222

[47] Levy C Naik J Giordano C Mandalia A O'Brien C Bhamidimarri KR. Hispanics with primary biliary cirrhosis are more likely to have features of autoimmune hepatitis and reduced response to ursodeoxycholic acid than non-Hispanics. Clin Gastroenterol Hepatol. 2014;12:1398–1405. 10.1016/j.cgh.2013.12.010 24361417

[48] Saturni S Bellini F Braido F Paggiaro P Sanduzzi A Scichilone N. Randomized controlled trials and real life studies. approaches and methodologies: A clinical point of view. Pulm Pharmacol Ther. 2014;27:129–138. 10.1016/j.pupt.2014.01.005 24468677

