# Supplementary material for: Sex and racial disparities in clinical outcomes and health resource use among patients with primary biliary cholangitis in the United States
Source: Hepatol Commun. 2026 Sep 28;10(10):e1030. doi: 10.1097/HC9.0000000000001030 (PMC13623276; doi:10.1097/HC9.0000000000001030)
Supplement: Supplementary file 1 [file hc9-10-e1030-s001.docx]

**Supplemental Digital Content (SDC)**

Sex and racial disparities in clinical outcomes and health resource use among patients with primary biliary cholangitis in the United States

Wilder JM, et al.

**SDC, Figure 1. Study design**

**Abbreviations:** HRU, health resource use; PBC, primary biliary cholangitis.

**SDC, Figure 2. Sample selection flowchart**

| Patients diagnosed with PBC on or after 01 January 2017  PBC diagnosis was defined as having ≥1 inpatient non-diagnostic claim or ≥2 distinct outpatient non-diagnostic claims with ICD-10-CM diagnosis code of PBC (K74.3)  PBC diagnosis date was defined as the first claim associated with ICD-10-CM diagnosis code of PBC | | | | | | | | | | | |
| --- | --- | --- | --- | --- | --- | --- | --- | --- | --- | --- | --- |
| N = 47,297 | | | | | | | | | | | |
|  |  |  |  |  |  |  |  |  |  |  |  |
| Patients aged ≥18 years at the time of PBC diagnosis | | | | | | | | | | | |
| N = 46,552 | | | | | | (98.4%) | | | | | |
|  |  |  |  |  |  |  |  |  |  |  |  |
| Patients had continuous enrollment for ≥12 months pre- and ≥1 month post-index | | | | | | | | | | | |
| Index date was defined as the date of initial PBC diagnosis | | | | | | | | | | | |
| N = 11,876 | | | | | | (25.5%) | | | | | |
|  |  |  |  |  |  |  |  |  |  |  |  |
| Patients had not received liver transplantation any time before the index date | | | | | | | | | | | |
| N = 11,836 | | | | | | (99.7%) | | | | | |
|  |  |  |  |  |  |  |  |  |  |  |  |
| Patients had not received OCA any time before the index date | | | | | | | | | | | |
| N = 11,765 | | | | | | (99.4%) | | | | | |
|  |  |  |  |  |  |  |  |  |  |  |  |
| Patients with race/ethnicity information available | | | | |  |  | Patients with sex information available | | | | |
| N = 9,554 | | | (81.2%) | |  |  | N = 11,591 | | (98.5%) | | |
|  |  |  |  |  |  |  |  |  |  |  |  |
| White | | | | |  |  | Female | | | | |
| N = 6,502 | | | (68.1%) | |  |  | N = 9,308 (80.3%) | | | | |
| Black | | | | |  |  | Male | | | | |
| N = 887 | | | (9.3%) | |  |  | N = 2,283 (19.7%) | | | | |
| Hispanic | | | | |  |  |  |  |  |  |  |
| N = 1,509 | | | (15.8%) | |  |  |  |  |  |  |  |
| Asian | | | | |  |  |  |  |  |  |  |
| N = 342 | | | (3.6%) | |  |  |  |  |  |  |  |
| Other | | | | |  |  |  |  |  |  |  |
| N = 314 | | | (3.3%) | |  |  |  |  |  |  |  |
|  |  |  |  |  |  |  |  |  |  |  |  |
| **Abbreviations:** ICD-10-CM, International Classification of Diseases, Tenth revision, Clinical Modification; OCA, obeticholic acid; PBC, primary biliary cholangitis. | | | | | | | | | | | |

**SDC, Table 1. Diagnosis codes for comorbidities, primary biliary cholangitis-related symptoms and conditions, and clinical outcomes of interest**

| **Condition** | **ICD-10 CM Codes** |
| --- | --- |
| **Charlson Comorbidity Index (CCI)^1^** |  |
| AIDS/HIV | B20.x-B22.x, B24.x |
| Any malignancy, including leukemia and lymphoma, except for malignant neoplasm of skin | C00.x-C26.x, C30.x-C34.x, C37.x-C41.x, C43.x, C45.x-C58.x, C60.x-C76.x, C81.x-C85.x, C88.x, C90.x-C97.x |
| Cerebrovascular disease | G45.x-G46.x, H34.0x, I60.x-I69.x |
| Chronic pulmonary disease | I27.8x-I27.9x, J40.x-J47.x, J60.x-J67.x, J68.4x, J70.1x, J70.3x |
| Congestive heart failure | I09.9x, I11.0x, I13.0x, I13.2x, I25.5x, I42.0x, I42.5x-I42.9x, I43.x, I50.x, P29.0x |
| Dementia | F00.x-F03.x, F05.1x, G30.x, G31.1x |
| Diabetes with chronic complications | E10.2x-E10.5x, E10.7x, E11.2x-E11.5x, E11.7x, E12.2x-E12.5x, E12.7x, E13.2x-E13.5x, E13.7x, E14.2x-E14.5x, E14.7x |
| Diabetes without chronic complications | E10.0x, E10.1x, E10.6x, E10.8x-E11.1x, E11.6x, E11.8x-E12.1x, E12.6x, E12.8x-E13.1x, E13.6x, E13.8x-E14.1x, E14.6x, E14.8x, E14.9x |
| Hemiplegia or paraplegia | G04.1x, G11.4x, G80.1x-G80.2x, G81.x-G82.x, G83.0x-G83.4x, G83.9x |
| Chronic hepatitis (or cirrhosis without portal hypertension) | B18.x, K70.0x-K70.3x, K70.9x, K71.3x-K71.5x, K71.7x, K73.x-K74.x, K76.0x, K76.2x-K76.4x, K76.8x-K76.9x, Z94.4x |
| Cirrhosis and portal hypertension with variceal bleeding history | I85.0x, I85.9x, I86.4x, I98.2x, K70.4x, K71.1x, K72.1x, K72.9x, K76.5x-K76.7x |
| Metastatic solid tumor | C77.x-C80.x |
| Myocardial infarction | I21.x, I22.x, I25.2x |
| Peptic ulcer disease | K25.x-K28.x |
| Peripheral vascular disease | I70.x-I71.x, I73.1x, I73.8x-I73.9x, I77.1x, I79.0x, I79.2x, K55.1x, K55.8x-K55.9x, Z95.8x-Z95.9x |
| Renal disease | I12.0x, I13.1x, N03.2x-N03.7x, N05.2x-N05.7x, N18.x-N19.x, N25.0x, Z49.0x-Z49.2x, Z94.0x, Z99.2x |
| Rheumatic disease | M05.x-M06.x, M31.5x, M32.x-M34.x, M35.1x, M35.3x, M36.0x |
| **PBC related symptoms/conditions and other comorbidities** | |
| Liver fibrosis | K74.00-K74.02 (Hepatic fibrosis)  K74.1 (Hepatic sclerosis)  K74.2 (Hepatic fibrosis with hepatic sclerosis) |
| Autoimmune hepatitis | K75.4 |
| Primary sclerosing cholangitis; | K83.01 |
| Chronic hepatitis NOS | K73.9 |
| Cholecystitis | K81.0-K81.2, K81.9 |
| Pruritus | L29.x |
|  |  |
| Fatigue | R53.82 (Chronic fatigue, unspecified)  R53.83 (Other fatigue) |
| Autoimmune thyroid disease | E06.3 |
| Raynaud’s syndrome | I73.00 - I73.01 |
| Sjögren’s syndrome | M35.0x |
| Systemic lupus erythematosus (SLE) | M32.x |
| Osteoporosis | M80.0x, M80.8x, M81.0, M81.6, M81.8 |
| Crohn's disease | K50.xx |
| Ulcerative colitis | K51.xx |
| Type 2 diabetes | E11.xx |
| Hypertension | I10 (Essential [primary] hypertension) I11 (Hypertensive heart disease)  I12 (Hypertensive chronic kidney disease)  I13 (Hypertensive heart and chronic kidney disease)  I15 (Secondary hypertension) I16 (Hypertensive crisis) |
| Dyslipidemia | E78.0 (Pure hypercholesterolemia)  E78.1 (Pure hyperglyceridemia)  E78.2 (Mixed hyperlipidemia)  E78.3 (Hyperchylomicronemia)  E78.4 (Other hyperlipidemia)  E78.5 (Hyperlipidemia, unspecified) |
| Gallstone disease (Cholelithiasis) | K80.xx |
| Inflammatory bowel disease | K52.x |
| **Outcomes and relevant conditions** |  |
| Cirrhosis | K74.6x (Other and unspecified cirrhosis of the liver) |
| Hepatic decompensation | R17 (Unspecified jaundice)  R18.0, R18.8 (Ascites)  K72.0x, K72.1x, K72.9x (End-stage liver disease)  K76.82 (Hepatic encephalopathy)  K76.7 (Hepatorenal syndrome)  I85.01, I85.11 (Bleeding esophageal varices) |
| Hepatocellular carcinoma | C22.0 |
| Clinically significant portal hypertension | K76.6 (Portal hypertension)  R16.1, R16.2, D73.81, D73.2 (Splenomegaly)  D69.6, D69.49, D69.59, D69.3 (Thrombocytopenia/low platelet count)  I85.x (Esophageal varices)  I86.4 (Gastric varices) |

**Abbreviations:** CCI, Charlson Comorbidity Index; ICD-9-CM, International Classification of Diseases, Ninth Revision, Clinical Modification; ICD-10-CM, International Classification of Diseases, Tenth Revision, Clinical Modification; HIV, human immunodeficiency virus; AIDS, Acquired immunodeficiency syndrome; NOS, not otherwise specified; SLE, Systemic lupus erythematosus. **Notes:** [1] CCI is calculated based on Quan et al. 2011. **Reference**: Quan H, Li B, Couris CM, Fushimi K, Graham P, Hider P, Januel JM, Sundararajan V. Updating and validating the Charlson comorbidity index and score for risk adjustment in hospital discharge abstracts using data from 6 countries. Am J Epidemiol. 2011 Mar 15;173(6):676-82. doi: 10.1093/aje/kwq433. Epub 2011 Feb 17. PMID: 21330339.

**SDC, Table 2. National Drug Code for primary biliary cholangitis treatments**

| **Generic name** | **NDC** | **Product name** | **GPI** | **GPI description** |
| --- | --- | --- | --- | --- |
| Ursodeoxycholic Acid (UDCA) | 80056014301 | RELTONE | 52100040000112 | Ursodiol Cap 200 MG |
|  | 72887014306 | URSODIOL | 52100040000112 | Ursodiol Cap 200 MG |
|  | 00023614501 | ACTIGALL | 52100040000120 | Ursodiol Cap 300 MG |
|  | 00078031905 | ACTIGALL | 52100040000120 | Ursodiol Cap 300 MG |
|  | 52544093001 | ACTIGALL | 52100040000120 | Ursodiol Cap 300 MG |
|  | 57267015330 | ACTIGALL | 52100040000120 | Ursodiol Cap 300 MG |
|  | 00093938001 | URSODIOL | 52100040000120 | Ursodiol Cap 300 MG |
|  | 00378173001 | URSODIOL | 52100040000120 | Ursodiol Cap 300 MG |
|  | 00527132601 | URSODIOL | 52100040000120 | Ursodiol Cap 300 MG |
|  | 00591315901 | URSODIOL | 52100040000120 | Ursodiol Cap 300 MG |
|  | 00781284801 | URSODIOL | 52100040000120 | Ursodiol Cap 300 MG |
|  | 00904622106 | URSODIOL | 52100040000120 | Ursodiol Cap 300 MG |
|  | 00904622160 | URSODIOL | 52100040000120 | Ursodiol Cap 300 MG |
|  | 00904622161 | URSODIOL | 52100040000120 | Ursodiol Cap 300 MG |
|  | 00904648360 | URSODIOL | 52100040000120 | Ursodiol Cap 300 MG |
|  | 10135054301 | URSODIOL | 52100040000120 | Ursodiol Cap 300 MG |
|  | 10702023701 | URSODIOL | 52100040000120 | Ursodiol Cap 300 MG |
|  | 24658078001 | URSODIOL | 52100040000120 | Ursodiol Cap 300 MG |
|  | 42291085010 | URSODIOL | 52100040000120 | Ursodiol Cap 300 MG |
|  | 42291085018 | URSODIOL | 52100040000120 | Ursodiol Cap 300 MG |
|  | 42385094601 | URSODIOL | 52100040000120 | Ursodiol Cap 300 MG |
|  | 42806050301 | URSODIOL | 52100040000120 | Ursodiol Cap 300 MG |
|  | 43063087101 | URSODIOL | 52100040000120 | Ursodiol Cap 300 MG |
|  | 43063087160 | URSODIOL | 52100040000120 | Ursodiol Cap 300 MG |
|  | 43063087193 | URSODIOL | 52100040000120 | Ursodiol Cap 300 MG |
|  | 50268079515 | URSODIOL | 52100040000120 | Ursodiol Cap 300 MG |
|  | 50268079611 | URSODIOL | 52100040000120 | Ursodiol Cap 300 MG |
|  | 50268079615 | URSODIOL | 52100040000120 | Ursodiol Cap 300 MG |
|  | 51079038301 | URSODIOL | 52100040000120 | Ursodiol Cap 300 MG |
|  | 51079038320 | URSODIOL | 52100040000120 | Ursodiol Cap 300 MG |
|  | 51079097001 | URSODIOL | 52100040000120 | Ursodiol Cap 300 MG |
|  | 51079097020 | URSODIOL | 52100040000120 | Ursodiol Cap 300 MG |
|  | 51224015150 | URSODIOL | 52100040000120 | Ursodiol Cap 300 MG |
|  | 52152006002 | URSODIOL | 52100040000120 | Ursodiol Cap 300 MG |
|  | 52152006003 | URSODIOL | 52100040000120 | Ursodiol Cap 300 MG |
|  | 54868503300 | URSODIOL | 52100040000120 | Ursodiol Cap 300 MG |
|  | 60429013801 | URSODIOL | 52100040000120 | Ursodiol Cap 300 MG |
|  | 60429066201 | URSODIOL | 52100040000120 | Ursodiol Cap 300 MG |
|  | 60687010001 | URSODIOL | 52100040000120 | Ursodiol Cap 300 MG |
|  | 60687010011 | URSODIOL | 52100040000120 | Ursodiol Cap 300 MG |
|  | 63629104801 | URSODIOL | 52100040000120 | Ursodiol Cap 300 MG |
|  | 64380084406 | URSODIOL | 52100040000120 | Ursodiol Cap 300 MG |
|  | 64980013901 | URSODIOL | 52100040000120 | Ursodiol Cap 300 MG |
|  | 68084021301 | URSODIOL | 52100040000120 | Ursodiol Cap 300 MG |
|  | 68084021311 | URSODIOL | 52100040000120 | Ursodiol Cap 300 MG |
|  | 69238154001 | URSODIOL | 52100040000120 | Ursodiol Cap 300 MG |
|  | 70710148301 | URSODIOL | 52100040000120 | Ursodiol Cap 300 MG |
|  | 71930001512 | URSODIOL | 52100040000120 | Ursodiol Cap 300 MG |
|  | 80056014401 | RELTONE | 52100040000130 | Ursodiol Cap 400 MG |
|  | 72887014406 | URSODIOL | 52100040000130 | Ursodiol Cap 400 MG |
|  | 00091078501 | URSO | 52100040000325 | Ursodiol Tab 250 MG |
|  | 00091078505 | URSO | 52100040000325 | Ursodiol Tab 250 MG |
|  | 00574078501 | URSO | 52100040000325 | Ursodiol Tab 250 MG |
|  | 12280030230 | URSO 250 | 52100040000325 | Ursodiol Tab 250 MG |
|  | 12280030260 | URSO 250 | 52100040000325 | Ursodiol Tab 250 MG |
|  | 58914078510 | URSO 250 | 52100040000325 | Ursodiol Tab 250 MG |
|  | 58914078550 | URSO 250 | 52100040000325 | Ursodiol Tab 250 MG |
|  | 00093536001 | URSODIOL | 52100040000325 | Ursodiol Tab 250 MG |
|  | 00115152401 | URSODIOL | 52100040000325 | Ursodiol Tab 250 MG |
|  | 00115993301 | URSODIOL | 52100040000325 | Ursodiol Tab 250 MG |
|  | 00591236801 | URSODIOL | 52100040000325 | Ursodiol Tab 250 MG |
|  | 00591299801 | URSODIOL | 52100040000325 | Ursodiol Tab 250 MG |
|  | 00904689004 | URSODIOL | 52100040000325 | Ursodiol Tab 250 MG |
|  | 49884041201 | URSODIOL | 52100040000325 | Ursodiol Tab 250 MG |
|  | 60687037825 | URSODIOL | 52100040000325 | Ursodiol Tab 250 MG |
|  | 60687037895 | URSODIOL | 52100040000325 | Ursodiol Tab 250 MG |
|  | 60687052711 | URSODIOL | 52100040000325 | Ursodiol Tab 250 MG |
|  | 64380091806 | URSODIOL | 52100040000325 | Ursodiol Tab 250 MG |
|  | 66993040502 | URSODIOL | 52100040000325 | Ursodiol Tab 250 MG |
|  | 68001022600 | URSODIOL | 52100040000325 | Ursodiol Tab 250 MG |
|  | 68001034300 | URSODIOL | 52100040000325 | Ursodiol Tab 250 MG |
|  | 68001037700 | URSODIOL | 52100040000325 | Ursodiol Tab 250 MG |
|  | 68084089225 | URSODIOL | 52100040000325 | Ursodiol Tab 250 MG |
|  | 68084089295 | URSODIOL | 52100040000325 | Ursodiol Tab 250 MG |
|  | 68462047301 | URSODIOL | 52100040000325 | Ursodiol Tab 250 MG |
|  | 70710112701 | URSODIOL | 52100040000325 | Ursodiol Tab 250 MG |
|  | 58914079010 | URSO FORTE | 52100040000350 | Ursodiol Tab 500 MG |
|  | 58914079050 | URSO FORTE | 52100040000350 | Ursodiol Tab 500 MG |
|  | 00093536101 | URSODIOL | 52100040000350 | Ursodiol Tab 500 MG |
|  | 00115152501 | URSODIOL | 52100040000350 | Ursodiol Tab 500 MG |
|  | 00115993401 | URSODIOL | 52100040000350 | Ursodiol Tab 500 MG |
|  | 00591236901 | URSODIOL | 52100040000350 | Ursodiol Tab 500 MG |
|  | 00591300501 | URSODIOL | 52100040000350 | Ursodiol Tab 500 MG |
|  | 49884041301 | URSODIOL | 52100040000350 | Ursodiol Tab 500 MG |
|  | 64380091906 | URSODIOL | 52100040000350 | Ursodiol Tab 500 MG |
|  | 66993040602 | URSODIOL | 52100040000350 | Ursodiol Tab 500 MG |
|  | 68001022700 | URSODIOL | 52100040000350 | Ursodiol Tab 500 MG |
|  | 68001034400 | URSODIOL | 52100040000350 | Ursodiol Tab 500 MG |
|  | 68001037800 | URSODIOL | 52100040000350 | Ursodiol Tab 500 MG |
|  | 68462047401 | URSODIOL | 52100040000350 | Ursodiol Tab 500 MG |
|  | 70710112801 | URSODIOL | 52100040000350 | Ursodiol Tab 500 MG |
|  | 38779198704 | URSODIOL | 96827657002900 | Ursodiol (Bulk) Powder |
|  | 38779198705 | URSODIOL | 96827657002900 | Ursodiol (Bulk) Powder |
|  | 38779198708 | URSODIOL | 96827657002900 | Ursodiol (Bulk) Powder |
|  | 49452808501 | URSODIOL | 96827657002900 | Ursodiol (Bulk) Powder |
|  | 49452808502 | URSODIOL | 96827657002900 | Ursodiol (Bulk) Powder |
|  | 51552090604 | URSODIOL | 96827657002900 | Ursodiol (Bulk) Powder |
|  | 51552090605 | URSODIOL | 96827657002900 | Ursodiol (Bulk) Powder |
|  | 51552090606 | URSODIOL | 96827657002900 | Ursodiol (Bulk) Powder |
|  | 51927290900 | URSODIOL | 96827657002900 | Ursodiol (Bulk) Powder |
|  | 52372063302 | URSODIOL | 96827657002900 | Ursodiol (Bulk) Powder |
|  | 62991218501 | URSODIOL | 96827657002900 | Ursodiol (Bulk) Powder |
|  | 62991218503 | URSODIOL | 96827657002900 | Ursodiol (Bulk) Powder |
|  | 62991218504 | URSODIOL | 96827657002900 | Ursodiol (Bulk) Powder |
|  | 62991279101 | URSODIOL | 96827657002900 | Ursodiol (Bulk) Powder |
|  | 62991279102 | URSODIOL | 96827657002900 | Ursodiol (Bulk) Powder |
|  | 62991279103 | URSODIOL | 96827657002900 | Ursodiol (Bulk) Powder |
|  | 62991279104 | URSODIOL | 96827657002900 | Ursodiol (Bulk) Powder |
|  | 63370032525 | URSODIOL | 96827657002900 | Ursodiol (Bulk) Powder |
|  | 63370032535 | URSODIOL | 96827657002900 | Ursodiol (Bulk) Powder |
| Obeticholic acid (OCA) | 69516000530 | OCALIVA | 52750060000320 | Obeticholic Acid Tab 5 MG |
|  | 69516001030 | OCALIVA | 52750060000330 | Obeticholic Acid Tab 10 MG |
| Gemfibrozil | 00005316031 | GEMFIBROZIL | 39200030000310 | Gemfibrozil Tab 600 MG |
|  | 00005316032 | GEMFIBROZIL | 39200030000310 | Gemfibrozil Tab 600 MG |
|  | 00047008420 | GEMFIBROZIL | 39200030000310 | Gemfibrozil Tab 600 MG |
|  | 00047008430 | GEMFIBROZIL | 39200030000310 | Gemfibrozil Tab 600 MG |
|  | 00093067005 | GEMFIBROZIL | 39200030000310 | Gemfibrozil Tab 600 MG |
|  | 00093067006 | GEMFIBROZIL | 39200030000310 | Gemfibrozil Tab 600 MG |
|  | 00093067019 | GEMFIBROZIL | 39200030000310 | Gemfibrozil Tab 600 MG |
|  | 00093067093 | GEMFIBROZIL | 39200030000310 | Gemfibrozil Tab 600 MG |
|  | 00115991113 | GEMFIBROZIL | 39200030000310 | Gemfibrozil Tab 600 MG |
|  | 00143913005 | GEMFIBROZIL | 39200030000310 | Gemfibrozil Tab 600 MG |
|  | 00143913060 | GEMFIBROZIL | 39200030000310 | Gemfibrozil Tab 600 MG |
|  | 00182195605 | GEMFIBROZIL | 39200030000310 | Gemfibrozil Tab 600 MG |
|  | 00182195689 | GEMFIBROZIL | 39200030000310 | Gemfibrozil Tab 600 MG |
|  | 00228255250 | GEMFIBROZIL | 39200030000310 | Gemfibrozil Tab 600 MG |
|  | 00364256605 | GEMFIBROZIL | 39200030000310 | Gemfibrozil Tab 600 MG |
|  | 00378051705 | GEMFIBROZIL | 39200030000310 | Gemfibrozil Tab 600 MG |
|  | 00378051791 | GEMFIBROZIL | 39200030000310 | Gemfibrozil Tab 600 MG |
|  | 00440756060 | GEMFIBROZIL | 39200030000310 | Gemfibrozil Tab 600 MG |
|  | 00440756092 | GEMFIBROZIL | 39200030000310 | Gemfibrozil Tab 600 MG |
|  | 00591045405 | GEMFIBROZIL | 39200030000310 | Gemfibrozil Tab 600 MG |
|  | 00591045460 | GEMFIBROZIL | 39200030000310 | Gemfibrozil Tab 600 MG |
|  | 00904537940 | GEMFIBROZIL | 39200030000310 | Gemfibrozil Tab 600 MG |
|  | 00904537952 | GEMFIBROZIL | 39200030000310 | Gemfibrozil Tab 600 MG |
|  | 00904537961 | GEMFIBROZIL | 39200030000310 | Gemfibrozil Tab 600 MG |
|  | 00904598840 | GEMFIBROZIL | 39200030000310 | Gemfibrozil Tab 600 MG |
|  | 00904598852 | GEMFIBROZIL | 39200030000310 | Gemfibrozil Tab 600 MG |
|  | 00904598861 | GEMFIBROZIL | 39200030000310 | Gemfibrozil Tab 600 MG |
|  | 00904773240 | GEMFIBROZIL | 39200030000310 | Gemfibrozil Tab 600 MG |
|  | 00904773252 | GEMFIBROZIL | 39200030000310 | Gemfibrozil Tab 600 MG |
|  | 16571078406 | GEMFIBROZIL | 39200030000310 | Gemfibrozil Tab 600 MG |
|  | 16571078450 | GEMFIBROZIL | 39200030000310 | Gemfibrozil Tab 600 MG |
|  | 16590096830 | GEMFIBROZIL | 39200030000310 | Gemfibrozil Tab 600 MG |
|  | 16714010102 | GEMFIBROZIL | 39200030000310 | Gemfibrozil Tab 600 MG |
|  | 16714010105 | GEMFIBROZIL | 39200030000310 | Gemfibrozil Tab 600 MG |
|  | 21695073260 | GEMFIBROZIL | 39200030000310 | Gemfibrozil Tab 600 MG |
|  | 23490562601 | GEMFIBROZIL | 39200030000310 | Gemfibrozil Tab 600 MG |
|  | 23490562602 | GEMFIBROZIL | 39200030000310 | Gemfibrozil Tab 600 MG |
|  | 24658013005 | GEMFIBROZIL | 39200030000310 | Gemfibrozil Tab 600 MG |
|  | 24658013018 | GEMFIBROZIL | 39200030000310 | Gemfibrozil Tab 600 MG |
|  | 24658013060 | GEMFIBROZIL | 39200030000310 | Gemfibrozil Tab 600 MG |
|  | 24658026005 | GEMFIBROZIL | 39200030000310 | Gemfibrozil Tab 600 MG |
|  | 24658026018 | GEMFIBROZIL | 39200030000310 | Gemfibrozil Tab 600 MG |
|  | 24658026060 | GEMFIBROZIL | 39200030000310 | Gemfibrozil Tab 600 MG |
|  | 24658026090 | GEMFIBROZIL | 39200030000310 | Gemfibrozil Tab 600 MG |
|  | 31722012805 | GEMFIBROZIL | 39200030000310 | Gemfibrozil Tab 600 MG |
|  | 31722012860 | GEMFIBROZIL | 39200030000310 | Gemfibrozil Tab 600 MG |
|  | 31722022501 | GEMFIBROZIL | 39200030000310 | Gemfibrozil Tab 600 MG |
|  | 31722022505 | GEMFIBROZIL | 39200030000310 | Gemfibrozil Tab 600 MG |
|  | 31722022560 | GEMFIBROZIL | 39200030000310 | Gemfibrozil Tab 600 MG |
|  | 42806026005 | GEMFIBROZIL | 39200030000310 | Gemfibrozil Tab 600 MG |
|  | 42806026060 | GEMFIBROZIL | 39200030000310 | Gemfibrozil Tab 600 MG |
|  | 43353009060 | GEMFIBROZIL | 39200030000310 | Gemfibrozil Tab 600 MG |
|  | 43353071460 | GEMFIBROZIL | 39200030000310 | Gemfibrozil Tab 600 MG |
|  | 43353075160 | GEMFIBROZIL | 39200030000310 | Gemfibrozil Tab 600 MG |
|  | 43353076260 | GEMFIBROZIL | 39200030000310 | Gemfibrozil Tab 600 MG |
|  | 43353077253 | GEMFIBROZIL | 39200030000310 | Gemfibrozil Tab 600 MG |
|  | 43353077260 | GEMFIBROZIL | 39200030000310 | Gemfibrozil Tab 600 MG |
|  | 43353077280 | GEMFIBROZIL | 39200030000310 | Gemfibrozil Tab 600 MG |
|  | 49999011430 | GEMFIBROZIL | 39200030000310 | Gemfibrozil Tab 600 MG |
|  | 49999011460 | GEMFIBROZIL | 39200030000310 | Gemfibrozil Tab 600 MG |
|  | 50268035011 | GEMFIBROZIL | 39200030000310 | Gemfibrozil Tab 600 MG |
|  | 50268035015 | GEMFIBROZIL | 39200030000310 | Gemfibrozil Tab 600 MG |
|  | 51079078701 | GEMFIBROZIL | 39200030000310 | Gemfibrozil Tab 600 MG |
|  | 51079078717 | GEMFIBROZIL | 39200030000310 | Gemfibrozil Tab 600 MG |
|  | 51079078719 | GEMFIBROZIL | 39200030000310 | Gemfibrozil Tab 600 MG |
|  | 51079078720 | GEMFIBROZIL | 39200030000310 | Gemfibrozil Tab 600 MG |
|  | 52189032024 | GEMFIBROZIL | 39200030000310 | Gemfibrozil Tab 600 MG |
|  | 52544045405 | GEMFIBROZIL | 39200030000310 | Gemfibrozil Tab 600 MG |
|  | 52544045460 | GEMFIBROZIL | 39200030000310 | Gemfibrozil Tab 600 MG |
|  | 52959093730 | GEMFIBROZIL | 39200030000310 | Gemfibrozil Tab 600 MG |
|  | 54569369500 | GEMFIBROZIL | 39200030000310 | Gemfibrozil Tab 600 MG |
|  | 54868235301 | GEMFIBROZIL | 39200030000310 | Gemfibrozil Tab 600 MG |
|  | 54868235302 | GEMFIBROZIL | 39200030000310 | Gemfibrozil Tab 600 MG |
|  | 55289041160 | GEMFIBROZIL | 39200030000310 | Gemfibrozil Tab 600 MG |
|  | 55289041193 | GEMFIBROZIL | 39200030000310 | Gemfibrozil Tab 600 MG |
|  | 57237016305 | GEMFIBROZIL | 39200030000310 | Gemfibrozil Tab 600 MG |
|  | 57237016360 | GEMFIBROZIL | 39200030000310 | Gemfibrozil Tab 600 MG |
|  | 57664011513 | GEMFIBROZIL | 39200030000310 | Gemfibrozil Tab 600 MG |
|  | 57664011586 | GEMFIBROZIL | 39200030000310 | Gemfibrozil Tab 600 MG |
|  | 57866654002 | GEMFIBROZIL | 39200030000310 | Gemfibrozil Tab 600 MG |
|  | 58016054060 | GEMFIBROZIL | 39200030000310 | Gemfibrozil Tab 600 MG |
|  | 58864071060 | GEMFIBROZIL | 39200030000310 | Gemfibrozil Tab 600 MG |
|  | 59762073705 | GEMFIBROZIL | 39200030000310 | Gemfibrozil Tab 600 MG |
|  | 60429008105 | GEMFIBROZIL | 39200030000310 | Gemfibrozil Tab 600 MG |
|  | 60429008118 | GEMFIBROZIL | 39200030000310 | Gemfibrozil Tab 600 MG |
|  | 60429008160 | GEMFIBROZIL | 39200030000310 | Gemfibrozil Tab 600 MG |
|  | 60429098118 | GEMFIBROZIL | 39200030000310 | Gemfibrozil Tab 600 MG |
|  | 60429098160 | GEMFIBROZIL | 39200030000310 | Gemfibrozil Tab 600 MG |
|  | 60505003400 | GEMFIBROZIL | 39200030000310 | Gemfibrozil Tab 600 MG |
|  | 60505003401 | GEMFIBROZIL | 39200030000310 | Gemfibrozil Tab 600 MG |
|  | 60505003402 | GEMFIBROZIL | 39200030000310 | Gemfibrozil Tab 600 MG |
|  | 60505003404 | GEMFIBROZIL | 39200030000310 | Gemfibrozil Tab 600 MG |
|  | 60505003406 | GEMFIBROZIL | 39200030000310 | Gemfibrozil Tab 600 MG |
|  | 60505003408 | GEMFIBROZIL | 39200030000310 | Gemfibrozil Tab 600 MG |
|  | 60687022401 | GEMFIBROZIL | 39200030000310 | Gemfibrozil Tab 600 MG |
|  | 60687022411 | GEMFIBROZIL | 39200030000310 | Gemfibrozil Tab 600 MG |
|  | 60809012255 | GEMFIBROZIL | 39200030000310 | Gemfibrozil Tab 600 MG |
|  | 60809012272 | GEMFIBROZIL | 39200030000310 | Gemfibrozil Tab 600 MG |
|  | 62269032029 | GEMFIBROZIL | 39200030000310 | Gemfibrozil Tab 600 MG |
|  | 62269032060 | GEMFIBROZIL | 39200030000310 | Gemfibrozil Tab 600 MG |
|  | 65862062405 | GEMFIBROZIL | 39200030000310 | Gemfibrozil Tab 600 MG |
|  | 65862062460 | GEMFIBROZIL | 39200030000310 | Gemfibrozil Tab 600 MG |
|  | 66336036760 | GEMFIBROZIL | 39200030000310 | Gemfibrozil Tab 600 MG |
|  | 67253074106 | GEMFIBROZIL | 39200030000310 | Gemfibrozil Tab 600 MG |
|  | 67253074150 | GEMFIBROZIL | 39200030000310 | Gemfibrozil Tab 600 MG |
|  | 67544009660 | GEMFIBROZIL | 39200030000310 | Gemfibrozil Tab 600 MG |
|  | 67544009680 | GEMFIBROZIL | 39200030000310 | Gemfibrozil Tab 600 MG |
|  | 67544013760 | GEMFIBROZIL | 39200030000310 | Gemfibrozil Tab 600 MG |
|  | 67544013780 | GEMFIBROZIL | 39200030000310 | Gemfibrozil Tab 600 MG |
|  | 67544043560 | GEMFIBROZIL | 39200030000310 | Gemfibrozil Tab 600 MG |
|  | 67544111160 | GEMFIBROZIL | 39200030000310 | Gemfibrozil Tab 600 MG |
|  | 67544114760 | GEMFIBROZIL | 39200030000310 | Gemfibrozil Tab 600 MG |
|  | 68084047301 | GEMFIBROZIL | 39200030000310 | Gemfibrozil Tab 600 MG |
|  | 68084047311 | GEMFIBROZIL | 39200030000310 | Gemfibrozil Tab 600 MG |
|  | 68382010005 | GEMFIBROZIL | 39200030000310 | Gemfibrozil Tab 600 MG |
|  | 68382010014 | GEMFIBROZIL | 39200030000310 | Gemfibrozil Tab 600 MG |
|  | 69097082103 | GEMFIBROZIL | 39200030000310 | Gemfibrozil Tab 600 MG |
|  | 69097082112 | GEMFIBROZIL | 39200030000310 | Gemfibrozil Tab 600 MG |
|  | 71610010753 | GEMFIBROZIL | 39200030000310 | Gemfibrozil Tab 600 MG |
|  | 71610010760 | GEMFIBROZIL | 39200030000310 | Gemfibrozil Tab 600 MG |
|  | 71610010780 | GEMFIBROZIL | 39200030000310 | Gemfibrozil Tab 600 MG |
|  | 71610020080 | GEMFIBROZIL | 39200030000310 | Gemfibrozil Tab 600 MG |
|  | 71610060153 | GEMFIBROZIL | 39200030000310 | Gemfibrozil Tab 600 MG |
|  | 71610060160 | GEMFIBROZIL | 39200030000310 | Gemfibrozil Tab 600 MG |
|  | 72865018605 | GEMFIBROZIL | 39200030000310 | Gemfibrozil Tab 600 MG |
|  | 72865018618 | GEMFIBROZIL | 39200030000310 | Gemfibrozil Tab 600 MG |
|  | 72865018660 | GEMFIBROZIL | 39200030000310 | Gemfibrozil Tab 600 MG |
|  | 75834013105 | GEMFIBROZIL | 39200030000310 | Gemfibrozil Tab 600 MG |
|  | 75834013118 | GEMFIBROZIL | 39200030000310 | Gemfibrozil Tab 600 MG |
|  | 75834013160 | GEMFIBROZIL | 39200030000310 | Gemfibrozil Tab 600 MG |
|  | 76282022505 | GEMFIBROZIL | 39200030000310 | Gemfibrozil Tab 600 MG |
|  | 76282022518 | GEMFIBROZIL | 39200030000310 | Gemfibrozil Tab 600 MG |
|  | 76282022560 | GEMFIBROZIL | 39200030000310 | Gemfibrozil Tab 600 MG |
|  | 00071073720 | LOPID | 39200030000310 | Gemfibrozil Tab 600 MG |
|  | 00071073730 | LOPID | 39200030000310 | Gemfibrozil Tab 600 MG |
|  | 54868141800 | LOPID | 39200030000310 | Gemfibrozil Tab 600 MG |
|  | 54868141801 | LOPID | 39200030000310 | Gemfibrozil Tab 600 MG |
|  | 38779037308 | GEMFIBROZIL | 39200030002900 | Gemfibrozil Powder |
|  | 49452326301 | GEMFIBROZIL | 39200030002900 | Gemfibrozil Powder |
|  | 51927264300 | GEMFIBROZIL | 39200030002900 | Gemfibrozil Powder |
| Fenofibrate | 61269021090 | FENOFIBRATE | 39200025000110 | Fenofibrate Cap 50 MG |
|  | 62559046090 | FENOFIBRATE | 39200025000110 | Fenofibrate Cap 50 MG |
|  | 66869013720 | LIPOFEN | 39200025000110 | Fenofibrate Cap 50 MG |
|  | 66869013730 | LIPOFEN | 39200025000110 | Fenofibrate Cap 50 MG |
|  | 61269021290 | FENOFIBRATE | 39200025000124 | Fenofibrate Cap 150 MG |
|  | 62559046190 | FENOFIBRATE | 39200025000124 | Fenofibrate Cap 150 MG |
|  | 62559030690 | LIPOFEN | 39200025000124 | Fenofibrate Cap 150 MG |
|  | 66869014720 | LIPOFEN | 39200025000124 | Fenofibrate Cap 150 MG |
|  | 66869014730 | LIPOFEN | 39200025000124 | Fenofibrate Cap 150 MG |
|  | 00115152210 | FENOFIBRATE | 39200025000308 | Fenofibrate Tab 40 MG |
|  | 00378439077 | FENOFIBRATE | 39200025000308 | Fenofibrate Tab 40 MG |
|  | 68682049090 | FENOFIBRATE | 39200025000308 | Fenofibrate Tab 40 MG |
|  | 52725049090 | FENOGLIDE | 39200025000308 | Fenofibrate Tab 40 MG |
|  | 59630049090 | FENOGLIDE | 39200025000308 | Fenofibrate Tab 40 MG |
|  | 68012049090 | FENOGLIDE | 39200025000308 | Fenofibrate Tab 40 MG |
|  | 00093206198 | FENOFIBRATE | 39200025000310 | Fenofibrate Tab 48 MG |
|  | 00093775598 | FENOFIBRATE | 39200025000310 | Fenofibrate Tab 48 MG |
|  | 00378306505 | FENOFIBRATE | 39200025000310 | Fenofibrate Tab 48 MG |
|  | 00378306577 | FENOFIBRATE | 39200025000310 | Fenofibrate Tab 48 MG |
|  | 16714073701 | FENOFIBRATE | 39200025000310 | Fenofibrate Tab 48 MG |
|  | 31722059590 | FENOFIBRATE | 39200025000310 | Fenofibrate Tab 48 MG |
|  | 43547043009 | FENOFIBRATE | 39200025000310 | Fenofibrate Tab 48 MG |
|  | 45802031575 | FENOFIBRATE | 39200025000310 | Fenofibrate Tab 48 MG |
|  | 51079059920 | FENOFIBRATE | 39200025000310 | Fenofibrate Tab 48 MG |
|  | 51407020490 | FENOFIBRATE | 39200025000310 | Fenofibrate Tab 48 MG |
|  | 60687061811 | FENOFIBRATE | 39200025000310 | Fenofibrate Tab 48 MG |
|  | 60687061821 | FENOFIBRATE | 39200025000310 | Fenofibrate Tab 48 MG |
|  | 62332036071 | FENOFIBRATE | 39200025000310 | Fenofibrate Tab 48 MG |
|  | 63304044805 | FENOFIBRATE | 39200025000310 | Fenofibrate Tab 48 MG |
|  | 63304044890 | FENOFIBRATE | 39200025000310 | Fenofibrate Tab 48 MG |
|  | 65862076890 | FENOFIBRATE | 39200025000310 | Fenofibrate Tab 48 MG |
|  | 68084063511 | FENOFIBRATE | 39200025000310 | Fenofibrate Tab 48 MG |
|  | 68084063521 | FENOFIBRATE | 39200025000310 | Fenofibrate Tab 48 MG |
|  | 68180036009 | FENOFIBRATE | 39200025000310 | Fenofibrate Tab 48 MG |
|  | 68180038809 | FENOFIBRATE | 39200025000310 | Fenofibrate Tab 48 MG |
|  | 68258610203 | FENOFIBRATE | 39200025000310 | Fenofibrate Tab 48 MG |
|  | 68382022816 | FENOFIBRATE | 39200025000310 | Fenofibrate Tab 48 MG |
|  | 68382026916 | FENOFIBRATE | 39200025000310 | Fenofibrate Tab 48 MG |
|  | 68682052501 | FENOFIBRATE | 39200025000310 | Fenofibrate Tab 48 MG |
|  | 69097045905 | FENOFIBRATE | 39200025000310 | Fenofibrate Tab 48 MG |
|  | 00074317390 | TRICOR | 39200025000310 | Fenofibrate Tab 48 MG |
|  | 00074612290 | TRICOR | 39200025000310 | Fenofibrate Tab 48 MG |
|  | 54868522400 | TRICOR | 39200025000310 | Fenofibrate Tab 48 MG |
|  | 68258600403 | TRICOR | 39200025000310 | Fenofibrate Tab 48 MG |
|  | 54868578400 | TRIGLIDE | 39200025000311 | Fenofibrate Tab 50 MG |
|  | 59630048030 | TRIGLIDE | 39200025000311 | Fenofibrate Tab 50 MG |
|  | 59630048090 | TRIGLIDE | 39200025000311 | Fenofibrate Tab 50 MG |
|  | 00115551110 | FENOFIBRATE | 39200025000312 | Fenofibrate Tab 54 MG |
|  | 00378710077 | FENOFIBRATE | 39200025000312 | Fenofibrate Tab 54 MG |
|  | 27241011603 | FENOFIBRATE | 39200025000312 | Fenofibrate Tab 54 MG |
|  | 35561034311 | FENOFIBRATE | 39200025000312 | Fenofibrate Tab 54 MG |
|  | 42291028990 | FENOFIBRATE | 39200025000312 | Fenofibrate Tab 54 MG |
|  | 42385093590 | FENOFIBRATE | 39200025000312 | Fenofibrate Tab 54 MG |
|  | 42858045445 | FENOFIBRATE | 39200025000312 | Fenofibrate Tab 54 MG |
|  | 43598090990 | FENOFIBRATE | 39200025000312 | Fenofibrate Tab 54 MG |
|  | 50268031211 | FENOFIBRATE | 39200025000312 | Fenofibrate Tab 54 MG |
|  | 50268031215 | FENOFIBRATE | 39200025000312 | Fenofibrate Tab 54 MG |
|  | 51138051830 | FENOFIBRATE | 39200025000312 | Fenofibrate Tab 54 MG |
|  | 51407009390 | FENOFIBRATE | 39200025000312 | Fenofibrate Tab 54 MG |
|  | 54868569700 | FENOFIBRATE | 39200025000312 | Fenofibrate Tab 54 MG |
|  | 62332053890 | FENOFIBRATE | 39200025000312 | Fenofibrate Tab 54 MG |
|  | 62332055490 | FENOFIBRATE | 39200025000312 | Fenofibrate Tab 54 MG |
|  | 63304090090 | FENOFIBRATE | 39200025000312 | Fenofibrate Tab 54 MG |
|  | 63629110001 | FENOFIBRATE | 39200025000312 | Fenofibrate Tab 54 MG |
|  | 68084082721 | FENOFIBRATE | 39200025000312 | Fenofibrate Tab 54 MG |
|  | 68084082725 | FENOFIBRATE | 39200025000312 | Fenofibrate Tab 54 MG |
|  | 68084082795 | FENOFIBRATE | 39200025000312 | Fenofibrate Tab 54 MG |
|  | 68180023109 | FENOFIBRATE | 39200025000312 | Fenofibrate Tab 54 MG |
|  | 68180036209 | FENOFIBRATE | 39200025000312 | Fenofibrate Tab 54 MG |
|  | 69367025709 | FENOFIBRATE | 39200025000312 | Fenofibrate Tab 54 MG |
|  | 70756021451 | FENOFIBRATE | 39200025000312 | Fenofibrate Tab 54 MG |
|  | 70756021490 | FENOFIBRATE | 39200025000312 | Fenofibrate Tab 54 MG |
|  | 57844069198 | LOFIBRA | 39200025000312 | Fenofibrate Tab 54 MG |
|  | 00074400990 | TRICOR | 39200025000312 | Fenofibrate Tab 54 MG |
|  | 54868459500 | TRICOR | 39200025000312 | Fenofibrate Tab 54 MG |
|  | 00115152310 | FENOFIBRATE | 39200025000322 | Fenofibrate Tab 120 MG |
|  | 00378439177 | FENOFIBRATE | 39200025000322 | Fenofibrate Tab 120 MG |
|  | 68682049590 | FENOFIBRATE | 39200025000322 | Fenofibrate Tab 120 MG |
|  | 52725049590 | FENOGLIDE | 39200025000322 | Fenofibrate Tab 120 MG |
|  | 54868611800 | FENOGLIDE | 39200025000322 | Fenofibrate Tab 120 MG |
|  | 59630049590 | FENOGLIDE | 39200025000322 | Fenofibrate Tab 120 MG |
|  | 68012049590 | FENOGLIDE | 39200025000322 | Fenofibrate Tab 120 MG |
|  | 00093206098 | FENOFIBRATE | 39200025000323 | Fenofibrate Tab 145 MG |
|  | 00093775698 | FENOFIBRATE | 39200025000323 | Fenofibrate Tab 145 MG |
|  | 00378306605 | FENOFIBRATE | 39200025000323 | Fenofibrate Tab 145 MG |
|  | 00378306677 | FENOFIBRATE | 39200025000323 | Fenofibrate Tab 145 MG |
|  | 00904716104 | FENOFIBRATE | 39200025000323 | Fenofibrate Tab 145 MG |
|  | 00904716161 | FENOFIBRATE | 39200025000323 | Fenofibrate Tab 145 MG |
|  | 16714073801 | FENOFIBRATE | 39200025000323 | Fenofibrate Tab 145 MG |
|  | 31722059690 | FENOFIBRATE | 39200025000323 | Fenofibrate Tab 145 MG |
|  | 43353024883 | FENOFIBRATE | 39200025000323 | Fenofibrate Tab 145 MG |
|  | 43547043109 | FENOFIBRATE | 39200025000323 | Fenofibrate Tab 145 MG |
|  | 45802013275 | FENOFIBRATE | 39200025000323 | Fenofibrate Tab 145 MG |
|  | 50268031111 | FENOFIBRATE | 39200025000323 | Fenofibrate Tab 145 MG |
|  | 50268031112 | FENOFIBRATE | 39200025000323 | Fenofibrate Tab 145 MG |
|  | 51079060820 | FENOFIBRATE | 39200025000323 | Fenofibrate Tab 145 MG |
|  | 51407020590 | FENOFIBRATE | 39200025000323 | Fenofibrate Tab 145 MG |
|  | 60687062921 | FENOFIBRATE | 39200025000323 | Fenofibrate Tab 145 MG |
|  | 62332036171 | FENOFIBRATE | 39200025000323 | Fenofibrate Tab 145 MG |
|  | 62332036190 | FENOFIBRATE | 39200025000323 | Fenofibrate Tab 145 MG |
|  | 63304044905 | FENOFIBRATE | 39200025000323 | Fenofibrate Tab 145 MG |
|  | 63304044990 | FENOFIBRATE | 39200025000323 | Fenofibrate Tab 145 MG |
|  | 65862076990 | FENOFIBRATE | 39200025000323 | Fenofibrate Tab 145 MG |
|  | 68084063625 | FENOFIBRATE | 39200025000323 | Fenofibrate Tab 145 MG |
|  | 68084063695 | FENOFIBRATE | 39200025000323 | Fenofibrate Tab 145 MG |
|  | 68180036102 | FENOFIBRATE | 39200025000323 | Fenofibrate Tab 145 MG |
|  | 68180036109 | FENOFIBRATE | 39200025000323 | Fenofibrate Tab 145 MG |
|  | 68180038902 | FENOFIBRATE | 39200025000323 | Fenofibrate Tab 145 MG |
|  | 68180038909 | FENOFIBRATE | 39200025000323 | Fenofibrate Tab 145 MG |
|  | 68382023016 | FENOFIBRATE | 39200025000323 | Fenofibrate Tab 145 MG |
|  | 68382027016 | FENOFIBRATE | 39200025000323 | Fenofibrate Tab 145 MG |
|  | 68682052801 | FENOFIBRATE | 39200025000323 | Fenofibrate Tab 145 MG |
|  | 69097045805 | FENOFIBRATE | 39200025000323 | Fenofibrate Tab 145 MG |
|  | 71610002783 | FENOFIBRATE | 39200025000323 | Fenofibrate Tab 145 MG |
|  | 00074318990 | TRICOR | 39200025000323 | Fenofibrate Tab 145 MG |
|  | 00074612390 | TRICOR | 39200025000323 | Fenofibrate Tab 145 MG |
|  | 12280032815 | TRICOR | 39200025000323 | Fenofibrate Tab 145 MG |
|  | 12280032830 | TRICOR | 39200025000323 | Fenofibrate Tab 145 MG |
|  | 12280032890 | TRICOR | 39200025000323 | Fenofibrate Tab 145 MG |
|  | 43353003283 | TRICOR | 39200025000323 | Fenofibrate Tab 145 MG |
|  | 43353003298 | TRICOR | 39200025000323 | Fenofibrate Tab 145 MG |
|  | 43353027230 | TRICOR | 39200025000323 | Fenofibrate Tab 145 MG |
|  | 43353027283 | TRICOR | 39200025000323 | Fenofibrate Tab 145 MG |
|  | 49999088530 | TRICOR | 39200025000323 | Fenofibrate Tab 145 MG |
|  | 49999088590 | TRICOR | 39200025000323 | Fenofibrate Tab 145 MG |
|  | 54569575000 | TRICOR | 39200025000323 | Fenofibrate Tab 145 MG |
|  | 54569575001 | TRICOR | 39200025000323 | Fenofibrate Tab 145 MG |
|  | 54868520300 | TRICOR | 39200025000323 | Fenofibrate Tab 145 MG |
|  | 54868520301 | TRICOR | 39200025000323 | Fenofibrate Tab 145 MG |
|  | 55289097330 | TRICOR | 39200025000323 | Fenofibrate Tab 145 MG |
|  | 58016004400 | TRICOR | 39200025000323 | Fenofibrate Tab 145 MG |
|  | 58016004430 | TRICOR | 39200025000323 | Fenofibrate Tab 145 MG |
|  | 58016004460 | TRICOR | 39200025000323 | Fenofibrate Tab 145 MG |
|  | 58016004490 | TRICOR | 39200025000323 | Fenofibrate Tab 145 MG |
|  | 67544103398 | TRICOR | 39200025000323 | Fenofibrate Tab 145 MG |
|  | 00115552202 | FENOFIBRATE | 39200025000325 | Fenofibrate Tab 160 MG |
|  | 00115552210 | FENOFIBRATE | 39200025000325 | Fenofibrate Tab 160 MG |
|  | 00179009180 | FENOFIBRATE | 39200025000325 | Fenofibrate Tab 160 MG |
|  | 00378710177 | FENOFIBRATE | 39200025000325 | Fenofibrate Tab 160 MG |
|  | 00904681004 | FENOFIBRATE | 39200025000325 | Fenofibrate Tab 160 MG |
|  | 00904712804 | FENOFIBRATE | 39200025000325 | Fenofibrate Tab 160 MG |
|  | 27241011703 | FENOFIBRATE | 39200025000325 | Fenofibrate Tab 160 MG |
|  | 27241011705 | FENOFIBRATE | 39200025000325 | Fenofibrate Tab 160 MG |
|  | 35561034411 | FENOFIBRATE | 39200025000325 | Fenofibrate Tab 160 MG |
|  | 35561034413 | FENOFIBRATE | 39200025000325 | Fenofibrate Tab 160 MG |
|  | 42291029030 | FENOFIBRATE | 39200025000325 | Fenofibrate Tab 160 MG |
|  | 42291029090 | FENOFIBRATE | 39200025000325 | Fenofibrate Tab 160 MG |
|  | 42385093605 | FENOFIBRATE | 39200025000325 | Fenofibrate Tab 160 MG |
|  | 42385093690 | FENOFIBRATE | 39200025000325 | Fenofibrate Tab 160 MG |
|  | 42858066045 | FENOFIBRATE | 39200025000325 | Fenofibrate Tab 160 MG |
|  | 43353000119 | FENOFIBRATE | 39200025000325 | Fenofibrate Tab 160 MG |
|  | 43353089819 | FENOFIBRATE | 39200025000325 | Fenofibrate Tab 160 MG |
|  | 43598091005 | FENOFIBRATE | 39200025000325 | Fenofibrate Tab 160 MG |
|  | 43598091090 | FENOFIBRATE | 39200025000325 | Fenofibrate Tab 160 MG |
|  | 50268031311 | FENOFIBRATE | 39200025000325 | Fenofibrate Tab 160 MG |
|  | 50268031315 | FENOFIBRATE | 39200025000325 | Fenofibrate Tab 160 MG |
|  | 51138051930 | FENOFIBRATE | 39200025000325 | Fenofibrate Tab 160 MG |
|  | 51407009490 | FENOFIBRATE | 39200025000325 | Fenofibrate Tab 160 MG |
|  | 54868566000 | FENOFIBRATE | 39200025000325 | Fenofibrate Tab 160 MG |
|  | 54868566001 | FENOFIBRATE | 39200025000325 | Fenofibrate Tab 160 MG |
|  | 60760090130 | FENOFIBRATE | 39200025000325 | Fenofibrate Tab 160 MG |
|  | 62332053990 | FENOFIBRATE | 39200025000325 | Fenofibrate Tab 160 MG |
|  | 62332053991 | FENOFIBRATE | 39200025000325 | Fenofibrate Tab 160 MG |
|  | 62332055590 | FENOFIBRATE | 39200025000325 | Fenofibrate Tab 160 MG |
|  | 62332055591 | FENOFIBRATE | 39200025000325 | Fenofibrate Tab 160 MG |
|  | 63304090105 | FENOFIBRATE | 39200025000325 | Fenofibrate Tab 160 MG |
|  | 63304090190 | FENOFIBRATE | 39200025000325 | Fenofibrate Tab 160 MG |
|  | 63629109903 | FENOFIBRATE | 39200025000325 | Fenofibrate Tab 160 MG |
|  | 68084032811 | FENOFIBRATE | 39200025000325 | Fenofibrate Tab 160 MG |
|  | 68084032821 | FENOFIBRATE | 39200025000325 | Fenofibrate Tab 160 MG |
|  | 68180023209 | FENOFIBRATE | 39200025000325 | Fenofibrate Tab 160 MG |
|  | 68180036309 | FENOFIBRATE | 39200025000325 | Fenofibrate Tab 160 MG |
|  | 69367025405 | FENOFIBRATE | 39200025000325 | Fenofibrate Tab 160 MG |
|  | 69367025409 | FENOFIBRATE | 39200025000325 | Fenofibrate Tab 160 MG |
|  | 69597034630 | FENOFIBRATE | 39200025000325 | Fenofibrate Tab 160 MG |
|  | 70199002730 | FENOFIBRATE | 39200025000325 | Fenofibrate Tab 160 MG |
|  | 70756021551 | FENOFIBRATE | 39200025000325 | Fenofibrate Tab 160 MG |
|  | 70756021590 | FENOFIBRATE | 39200025000325 | Fenofibrate Tab 160 MG |
|  | 71205094955 | FENOFIBRATE | 39200025000325 | Fenofibrate Tab 160 MG |
|  | 71205094990 | FENOFIBRATE | 39200025000325 | Fenofibrate Tab 160 MG |
|  | 57844069298 | LOFIBRA | 39200025000325 | Fenofibrate Tab 160 MG |
|  | 00074401390 | TRICOR | 39200025000325 | Fenofibrate Tab 160 MG |
|  | 54868459700 | TRICOR | 39200025000325 | Fenofibrate Tab 160 MG |
|  | 59630048530 | TRIGLIDE | 39200025000325 | Fenofibrate Tab 160 MG |
|  | 59630048590 | TRIGLIDE | 39200025000325 | Fenofibrate Tab 160 MG |
|  | 70199001330 | TRIGLIDE | 39200025000325 | Fenofibrate Tab 160 MG |
|  | 27437010706 | ANTARA | 39200025100103 | Fenofibrate Micronized Cap 30 MG |
|  | 68180074506 | FENOFIBRATE MICRONIZED | 39200025100103 | Fenofibrate Micronized Cap 30 MG |
|  | 27437010906 | ANTARA | 39200025100104 | Fenofibrate Micronized Cap 43 MG |
|  | 65726040110 | ANTARA | 39200025100104 | Fenofibrate Micronized Cap 43 MG |
|  | 65726040125 | ANTARA | 39200025100104 | Fenofibrate Micronized Cap 43 MG |
|  | 67707004330 | ANTARA | 39200025100104 | Fenofibrate Micronized Cap 43 MG |
|  | 00378608893 | FENOFIBRATE | 39200025100104 | Fenofibrate Micronized Cap 43 MG |
|  | 55111034930 | FENOFIBRATE | 39200025100104 | Fenofibrate Micronized Cap 43 MG |
|  | 60505312003 | FENOFIBRATE | 39200025100104 | Fenofibrate Micronized Cap 43 MG |
|  | 63304044330 | FENOFIBRATE | 39200025100104 | Fenofibrate Micronized Cap 43 MG |
|  | 68180013006 | FENOFIBRATE | 39200025100104 | Fenofibrate Micronized Cap 43 MG |
|  | 13668044001 | FENOFIBRATE | 39200025100107 | Fenofibrate Micronized Cap 67 MG |
|  | 62332008431 | FENOFIBRATE | 39200025100107 | Fenofibrate Micronized Cap 67 MG |
|  | 00115051101 | FENOFIBRATE MICRONIZED | 39200025100107 | Fenofibrate Micronized Cap 67 MG |
|  | 00378862877 | FENOFIBRATE MICRONIZED | 39200025100107 | Fenofibrate Micronized Cap 67 MG |
|  | 16714073902 | FENOFIBRATE MICRONIZED | 39200025100107 | Fenofibrate Micronized Cap 67 MG |
|  | 27241011804 | FENOFIBRATE MICRONIZED | 39200025100107 | Fenofibrate Micronized Cap 67 MG |
|  | 42858006701 | FENOFIBRATE MICRONIZED | 39200025100107 | Fenofibrate Micronized Cap 67 MG |
|  | 43975044410 | FENOFIBRATE MICRONIZED | 39200025100107 | Fenofibrate Micronized Cap 67 MG |
|  | 51407009090 | FENOFIBRATE MICRONIZED | 39200025100107 | Fenofibrate Micronized Cap 67 MG |
|  | 68462058001 | FENOFIBRATE MICRONIZED | 39200025100107 | Fenofibrate Micronized Cap 67 MG |
|  | 69097089407 | FENOFIBRATE MICRONIZED | 39200025100107 | Fenofibrate Micronized Cap 67 MG |
|  | 69367027001 | FENOFIBRATE MICRONIZED | 39200025100107 | Fenofibrate Micronized Cap 67 MG |
|  | 57844032201 | LOFIBRA | 39200025100107 | Fenofibrate Micronized Cap 67 MG |
|  | 00074434290 | TRICOR | 39200025100107 | Fenofibrate Micronized Cap 67 MG |
|  | 27437010806 | ANTARA | 39200025100111 | Fenofibrate Micronized Cap 90 MG |
|  | 68180074606 | FENOFIBRATE MICRONIZED | 39200025100111 | Fenofibrate Micronized Cap 90 MG |
|  | 27437011001 | ANTARA | 39200025100114 | Fenofibrate Micronized Cap 130 M |
|  | 27437011006 | ANTARA | 39200025100114 | Fenofibrate Micronized Cap 130 M |
|  | 54868549800 | ANTARA | 39200025100114 | Fenofibrate Micronized Cap 130 M |
|  | 65726040310 | ANTARA | 39200025100114 | Fenofibrate Micronized Cap 130 M |
|  | 65726040325 | ANTARA | 39200025100114 | Fenofibrate Micronized Cap 130 M |
|  | 67707013030 | ANTARA | 39200025100114 | Fenofibrate Micronized Cap 130 M |
|  | 67707013099 | ANTARA | 39200025100114 | Fenofibrate Micronized Cap 130 M |
|  | 00378608977 | FENOFIBRATE | 39200025100114 | Fenofibrate Micronized Cap 130 M |
|  | 00378608993 | FENOFIBRATE | 39200025100114 | Fenofibrate Micronized Cap 130 M |
|  | 42291029290 | FENOFIBRATE | 39200025100114 | Fenofibrate Micronized Cap 130 M |
|  | 55111039530 | FENOFIBRATE | 39200025100114 | Fenofibrate Micronized Cap 130 M |
|  | 55111039590 | FENOFIBRATE | 39200025100114 | Fenofibrate Micronized Cap 130 M |
|  | 60505312103 | FENOFIBRATE | 39200025100114 | Fenofibrate Micronized Cap 130 M |
|  | 60505312109 | FENOFIBRATE | 39200025100114 | Fenofibrate Micronized Cap 130 M |
|  | 63304044430 | FENOFIBRATE | 39200025100114 | Fenofibrate Micronized Cap 130 M |
|  | 63304044490 | FENOFIBRATE | 39200025100114 | Fenofibrate Micronized Cap 130 M |
|  | 68180013106 | FENOFIBRATE | 39200025100114 | Fenofibrate Micronized Cap 130 M |
|  | 00093801101 | FENOFIBRATE | 39200025100115 | Fenofibrate Micronized Cap 134 M |
|  | 13668043901 | FENOFIBRATE | 39200025100115 | Fenofibrate Micronized Cap 134 M |
|  | 62332008531 | FENOFIBRATE | 39200025100115 | Fenofibrate Micronized Cap 134 M |
|  | 00115052201 | FENOFIBRATE MICRONIZED | 39200025100115 | Fenofibrate Micronized Cap 134 M |
|  | 00115052202 | FENOFIBRATE MICRONIZED | 39200025100115 | Fenofibrate Micronized Cap 134 M |
|  | 00378862905 | FENOFIBRATE MICRONIZED | 39200025100115 | Fenofibrate Micronized Cap 134 M |
|  | 00378862977 | FENOFIBRATE MICRONIZED | 39200025100115 | Fenofibrate Micronized Cap 134 M |
|  | 16714074002 | FENOFIBRATE MICRONIZED | 39200025100115 | Fenofibrate Micronized Cap 134 M |
|  | 27241011904 | FENOFIBRATE MICRONIZED | 39200025100115 | Fenofibrate Micronized Cap 134 M |
|  | 42858013401 | FENOFIBRATE MICRONIZED | 39200025100115 | Fenofibrate Micronized Cap 134 M |
|  | 43975030510 | FENOFIBRATE MICRONIZED | 39200025100115 | Fenofibrate Micronized Cap 134 M |
|  | 51407009190 | FENOFIBRATE MICRONIZED | 39200025100115 | Fenofibrate Micronized Cap 134 M |
|  | 54868599401 | FENOFIBRATE MICRONIZED | 39200025100115 | Fenofibrate Micronized Cap 134 M |
|  | 68084083532 | FENOFIBRATE MICRONIZED | 39200025100115 | Fenofibrate Micronized Cap 134 M |
|  | 68084083533 | FENOFIBRATE MICRONIZED | 39200025100115 | Fenofibrate Micronized Cap 134 M |
|  | 68462058101 | FENOFIBRATE MICRONIZED | 39200025100115 | Fenofibrate Micronized Cap 134 M |
|  | 69097089507 | FENOFIBRATE MICRONIZED | 39200025100115 | Fenofibrate Micronized Cap 134 M |
|  | 69367026801 | FENOFIBRATE MICRONIZED | 39200025100115 | Fenofibrate Micronized Cap 134 M |
|  | 57844032301 | LOFIBRA | 39200025100115 | Fenofibrate Micronized Cap 134 M |
|  | 00074644790 | TRICOR | 39200025100115 | Fenofibrate Micronized Cap 134 M |
|  | 00093801201 | FENOFIBRATE | 39200025100130 | Fenofibrate Micronized Cap 200 M |
|  | 13668043801 | FENOFIBRATE | 39200025100130 | Fenofibrate Micronized Cap 200 M |
|  | 59651020301 | FENOFIBRATE | 39200025100130 | Fenofibrate Micronized Cap 200 M |
|  | 62332008631 | FENOFIBRATE | 39200025100130 | Fenofibrate Micronized Cap 200 M |
|  | 00115053301 | FENOFIBRATE MICRONIZED | 39200025100130 | Fenofibrate Micronized Cap 200 M |
|  | 00115053302 | FENOFIBRATE MICRONIZED | 39200025100130 | Fenofibrate Micronized Cap 200 M |
|  | 00378863005 | FENOFIBRATE MICRONIZED | 39200025100130 | Fenofibrate Micronized Cap 200 M |
|  | 00378863077 | FENOFIBRATE MICRONIZED | 39200025100130 | Fenofibrate Micronized Cap 200 M |
|  | 16714074102 | FENOFIBRATE MICRONIZED | 39200025100130 | Fenofibrate Micronized Cap 200 M |
|  | 27241012004 | FENOFIBRATE MICRONIZED | 39200025100130 | Fenofibrate Micronized Cap 200 M |
|  | 42858020001 | FENOFIBRATE MICRONIZED | 39200025100130 | Fenofibrate Micronized Cap 200 M |
|  | 43975030610 | FENOFIBRATE MICRONIZED | 39200025100130 | Fenofibrate Micronized Cap 200 M |
|  | 54868557500 | FENOFIBRATE MICRONIZED | 39200025100130 | Fenofibrate Micronized Cap 200 M |
|  | 54868557501 | FENOFIBRATE MICRONIZED | 39200025100130 | Fenofibrate Micronized Cap 200 M |
|  | 54868557502 | FENOFIBRATE MICRONIZED | 39200025100130 | Fenofibrate Micronized Cap 200 M |
|  | 68084032911 | FENOFIBRATE MICRONIZED | 39200025100130 | Fenofibrate Micronized Cap 200 M |
|  | 68084032921 | FENOFIBRATE MICRONIZED | 39200025100130 | Fenofibrate Micronized Cap 200 M |
|  | 68462058201 | FENOFIBRATE MICRONIZED | 39200025100130 | Fenofibrate Micronized Cap 200 M |
|  | 69097089607 | FENOFIBRATE MICRONIZED | 39200025100130 | Fenofibrate Micronized Cap 200 M |
|  | 69367026901 | FENOFIBRATE MICRONIZED | 39200025100130 | Fenofibrate Micronized Cap 200 M |
|  | 54868551200 | LOFIBRA | 39200025100130 | Fenofibrate Micronized Cap 200 M |
|  | 57844032401 | LOFIBRA | 39200025100130 | Fenofibrate Micronized Cap 200 M |
|  | 00074641590 | TRICOR | 39200025100130 | Fenofibrate Micronized Cap 200 M |

**Abbreviations:** GPC, Generic Product Identifier; NDC, National Drug Code; OCA, obeticholic acid; PBC, primary biliary cholangitis; UDCA, ursodeoxycholic acid.

**SDC, Table 3. Procedure codes for primary biliary cholangitis treatments and primary biliary cholangitis-related tests**

| **Treatment/Procedure** | **CPT/HCPCS Codes^1^** | **ICD-10 PCS Codes** |
| --- | --- | --- |
| **PBC treatment** |  |  |
| Liver transplant | 47135 (liver allotransplantation) | 0FY00Z0 (allogenic)  0FY00Z1 (syngeneic)  0FY00Z2 (zooplastic) |
| **PBC-related tests** |  |  |
| Antimitochondrial antibody (AMA test) | 86381 (mitochondrial antibody each) | NA |
| Aspartate aminotransferase (AST) test | 84450 (transferase; aspartate amino (AST)) | NA |
| Alanine aminotransferase (ALT) test | 84460 (transferase; alanine amino (ALT)) | NA |
| Alkaline phosphatase (ALP) test | 84075 (phosphatase, alkaline)  84078 (phosphatase, alkaline; heat stable)  84080 (phosphatase, alkaline; isoenzymes) | NA |
| Serum bilirubin test | 82247 (bilirubin, total)  82248 (bilirubin, direct) | NA |
| FibroScan (transient elastography) | 91200 (liver elastography, mechanically induced shear wave [e.g., vibration], without imaging, with interpretation and report) | NA |
| Liver biopsy | 47000 (biopsy of liver, needle; percutaneous)  47001 (biopsy of liver, needle; when done for indicated purpose at time of other major procedure)  47100 (biopsy of liver, wedge)  43261 (biopsy of gallbladder, pancreatic, liver, and bile ducts using a flexible endoscope) | 0FB00ZX (excision of liver, open approach, diagnostic)  0FB03ZX (excision of liver, percutaneous approach, diagnostic)  0FB04ZX (excision of liver, percutaneous endoscopic approach, diagnostic)  0FB10ZX (excision of right lobe liver, open approach, diagnostic)  0FB13ZX (excision of right lobe liver, percutaneous approach, diagnostic)  0FB14ZX (excision of right lobe liver, percutaneous endoscopic approach, diagnostic)  0FB20ZX (excision of left lobe liver, open approach, diagnostic)  0FB23ZX (excision of left lobe liver, percutaneous approach, diagnostic)  0FB24ZX (excision of left lobe liver, percutaneous endoscopic approach, diagnostic) |

**Abbreviations:** CPT, Current Procedural Terminology; HCPCS, Healthcare Common Procedure Coding System; ICD-10-PCS, International Classification of Diseases, Tenth Revision, Procedure Coding System; NA, not applicable; PBC, primary biliary cholangitis. **Notes:** [1] NA denotes ICD-10 PCS Codes cannot be found for the procedure.

**SDC, Table 4. Summary of unadjusted incidence rate ratios for all-cause healthcare resource use (per-person-per-year) during the post-index period (race/ethnicity analysis)**

a) **Number of medical visits**

|  | **Black vs White** | **Hispanic White** | **Asian vs White** | **Others vs White** |
| --- | --- | --- | --- | --- |
| **Unadjusted IRRs (95% CI)^1^** | |  |  |  |
| OP visits | 1.19 (1.13, 1.26)* | 1.11 (1.06, 1.16)* | 0.93 (0.85, 1.01) | 1.03 (0.94, 1.13) |
| ER visits | 2.17 (1.94, 2.43)* | 1.48 (1.35, 1.63)* | 0.67 (0.55, 0.81)* | 1.21 (1.00, 1.47)* |
| IP admissions | 1.56 (1.33, 1.83)* | 0.99 (0.86, 1.13) | 0.75 (0.58, 0.98)* | 0.95 (0.72, 1.24) |
| IP length of stay (days) | 1.61 (1.28, 2.03)* | 0.98 (0.81, 1.18) | 0.75 (0.52, 1.07) | 0.66 (0.45, 0.97)* |

b) **Number of liver function lab tests**

|  | **Black vs White** | **Hispanic vs White** | **Asian vs White** | **Others vs White** |
| --- | --- | --- | --- | --- |
| **Unadjusted IRRs (95% CI)^1^** | |  |  |  |
| ALP tests | 1.09 (0.96, 1.23) | 0.87 (0.79, 0.96)* | 1.08 (0.90, 1.30) | 0.81 (0.66, 0.99)* |
| AST tests | 1.09 (0.96, 1.24) | 0.86 (0.78, 0.96)* | 1.07 (0.89, 1.29) | 0.78 (0.63, 0.96)* |
| ALT tests | 1.10 (0.97, 1.25) | 0.85 (0.77, 0.94)* | 1.03 (0.85, 1.24) | 0.76 (0.62, 0.94)* |
| Serum bilirubin tests | 1.15 (1.02, 1.29)* | 0.90 (0.82, 0.99)* | 0.97 (0.82, 1.16) | 0.85 (0.70, 1.03) |

**Abbreviations:** ALP, alkaline phosphatase; ALT, alanine aminotransferase; AST, aspartate aminotransferase; CI, confidence interval; ER, emergency room; HRU, healthcare resource use; IP, inpatient; IRR, incidence rate ratio; OP, outpatient. **Note**: [1] Unadjusted IRRs and p-values were estimated using univariable generalized linear models with a negative binomial distribution and a log-link function. Person-time is included in the offset to model the rate. [2]* Indicates a statistically significant difference (p<0.05).

**SDC, Table 5. Summary of unadjusted hazard ratios for time to adverse clinical outcomes, time to first primary biliary cholangitis treatment, and time to first specialist visit during the post-index period by race/ethnicity based on Cox proportional hazard models**

|  | **Black** | **Hispanic** | **Asian** | **Others** |
| --- | --- | --- | --- | --- |
|  | **HR (95% CI)** | **HR (95% CI)** | **HR (95% CI)** | **HR (95% CI)** |
| **Adverse clinical outcomes** |  |  |  |  |
| Hepatic decompensation | 1.31 (1.07, 1.59)* | 0.94 (0.79, 1.13) | 0.82 (0.57, 1.18) | 0.77 (0.51, 1.15) |
| IP due to hepatic decompensation | 1.50 (1.17, 1.92)* | 1.06 (0.84, 1.32) | 0.86 (0.54, 1.38) | 0.67 (0.38, 1.19) |
| Liver transplant | 1.75 (1.19, 2.57)* | 0.98 (0.67, 1.45) | 1.63 (0.90, 2.93) | 0.96 (0.43, 2.18) |
| Cirrhosis | 1.11 (0.94, 1.32) | 1.16 (1.02, 1.33)* | 0.91 (0.69, 1.19) | 0.67 (0.47, 0.94)* |
| Clinically significant portal hypertension | 1.11 (0.92, 1.34) | 1.00 (0.85, 1.17) | 1.04 (0.78, 1.39) | 0.74 (0.51, 1.08) |
| HCC | 1.09 (0.62, 1.90) | 1.35 (0.90, 2.03) | 1.34 (0.62, 2.88) | 1.14 (0.46, 2.79) |
| End-stage liver disease | 1.22 (0.97, 1.54) | 0.97 (0.80, 1.19) | 0.98 (0.67, 1.43) | 0.95 (0.63, 1.44) |
| All-cause death | 1.09 (0.89, 1.33) | 0.71 (0.59, 0.86)* | 0.40 (0.24, 0.65)* | 1.07 (0.76, 1.50) |
| Earliest of any clinical outcomes | 1.05 (0.89, 1.23) | 1.07 (0.95, 1.22) | 0.84 (0.65, 1.09) | 0.71 (0.53, 0.97)* |

**Time to first PBC treatment**

| First PBC treatment | 0.86 (0.79, 0.94)* | 0.93 (0.87, 1.00)* | 0.97 (0.85, 1.10) | 1.00 (0.88, 1.14) |
| --- | --- | --- | --- | --- |
| **Time to first specialist visit** |  |  |  |  |
| First specialist visit | 0.99 (0.85, 1.16) | 1.00 (0.89, 1.13) | 1.12 (0.88, 1.42) | 0.94, (0.73, 1.20) |

**Abbreviations:** CI, confidence interval; HCC, hepatocellular carcinoma; HR, hazard ratio; IP, inpatient; PBC, primary biliary cholangitis. **Note:** [1] * Indicates a statistically significant difference (p<0.05).

**SDC, Table 6. Summary of unadjusted incidence rate ratios for all-cause healthcare resource use (per-patient-per-year) during the post-index period (sex analysis)**

a) **Number of medical visits**

|  | **Male vs Female** |
| --- | --- |
| **Unadjusted IRRs (95% CI)^1^** |  |
| OP visits | 1.17 (1.13, 1.22)* |
| ER visits | 1.29 (1.20, 1.40)* |
| IP admissions | 2.72 (2.44, 3.02)* |
| IP length of stay (days) | 2.52 (2.15, 2.94)* |

b) **Number of liver function lab tests**

|  | **Male vs Female** |
| --- | --- |
| **Unadjusted IRRs (95% CI)^1^** |  |
| ALP tests | 1.43 (1.32, 1.54)* |
| AST tests | 1.42 (1.31, 1.54)* |
| ALT tests | 1.43 (1.32, 1.55)* |
| Serum bilirubin tests | 1.68 (1.56, 1.80)* |

**Abbreviations:** ALP, alkaline phosphatase; ALT, alanine aminotransferase; AST, aspartate aminotransferase; CI, confidence interval; ER, emergency room; HRU, healthcare resource use; IP, inpatient; IRR, incidence rate ratio; PPPY, per-patient-per-year; OP, outpatient. **Note:** [1] Unadjusted IRRs and p-values were estimated using univariable generalized linear models with a negative binomial distribution and a log-link function. [2] * Indicates a statistically significant difference (p<0.05).

**SDC, Table 7. Summary of unadjusted hazard ratios for time to adverse clinical outcomes, time to first primary biliary cholangitis treatment, and time to first specialist visit during the post-index period by sex based on Cox proportional hazard models**

|  | **Male vs Female** |
| --- | --- |
|  | **N = 2,283** |
| **Adverse clinical outcomes** |  |
| Hepatic decompensation | 2.50 (2.20, 2.83)* |
| IP due to hepatic decompensation | 2.99 (2.55, 3.50)* |
| Liver transplant | 5.70 (4.51, 7.19)* |
| Cirrhosis | 1.45 (1.29, 1.63)* |
| Clinically significant portal hypertension | 2.18 (1.93, 2.46)* |
| HCC | 2.72 (2.02, 3.67)* |
| End-stage liver disease | 2.77 (2.42, 3.16)* |
| All-cause death | 2.51 (2.22, 2.83)* |
| Earliest of any clinical outcomes | 1.61 (1.45, 1.80)* |
| **Time to first PBC treatment** |  |
| First treatment | 0.65 (0.62, 0.69)* |
| **Time to first specialist visit** |  |
| First specialist visit | 0.87 (0.78, 0.97)* |

**Abbreviations:** IP, inpatient; HCC, hepatocellular carcinoma; HR, hazard ratio; PBC, primary biliary cholangitis. **Note**: [1] * Indicates a statistically significant difference (p<0.05).

**SDC, Plain Language Summary**

Primary biliary cholangitis is a rare liver disease that worsens over time and can be life-threatening. It is important to diagnose and treat primary biliary cholangitis early to help slow or stop disease progression. The likelihood of developing liver‑related complications and the way patients use healthcare services may vary depending on their race, ethnicity, or sex. Acting on these differences can help bring about changes so that more patients have timely access to treatment to improve their health. The current study used United States health insurance records to understand how patients of different races and ethnicities (White, Black, Hispanic, Asian, or Other) and sexes (male or female) used healthcare services before and after a diagnosis of primary biliary cholangitis, and how the risks of developing liver complications differ across these groups. Before diagnosis, Black and Hispanic patients experienced more severe symptoms than White patients; men also had more severe symptoms compared to women. Despite having worse symptoms, these groups were less likely to receive treatment. After diagnosis, Black and Hispanic patients were more likely to have severe health complications than White patients, while men generally faced higher risks for all health complications than women. Black and Hispanic patients had lower timely treatment rates and poorer responses than White patients, with a similar result observed for men compared to women. These results show that healthcare providers should pay more attention to differences in treatment decisions and outcomes by race/ethnicity and sex to ensure all patients receive care at the right time.
